# Exploring the evolution of protein function in Archaea

**DOI:** 10.1186/1471-2148-12-75

**Published:** 2012-05-30

**Authors:** Alexander Goncearenco, Igor N Berezovsky

**Affiliations:** 1 Computational Biology Unit, Uni Research, University of Bergen, N-5008, Bergen, Norway; 2 Department of Informatics, University of Bergen, N-5008, Bergen, Norway

**Keywords:** Protein function, Evolution, Archaea, Elementary functional loops, Functional domains/folds

## Abstract

**Background:**

Despite recent progress in studies of the evolution of protein function, the questions what were the first functional protein domains and what were their basic building blocks remain unresolved. Previously, we introduced the concept of elementary functional loops (EFLs), which are the functional units of enzymes that provide elementary reactions in biochemical transformations. They are presumably descendants of primordial catalytic peptides.

**Results:**

We analyzed distant evolutionary connections between protein functions in Archaea based on the EFLs comprising them. We show examples of the involvement of EFLs in new functional domains, as well as reutilization of EFLs and functional domains in building multidomain structures and protein complexes.

**Conclusions:**

Our analysis of the archaeal superkingdom yields the dominating mechanisms in different periods of protein evolution, which resulted in several levels of the organization of biochemical function. First, functional domains emerged as combinations of prebiotic peptides with the very basic functions, such as nucleotide/phosphate and metal cofactor binding. Second, domain recombination brought to the evolutionary scene the multidomain proteins and complexes. Later, reutilization and *de novo* design of functional domains and elementary functional loops complemented evolution of protein function.

## Background

Protein evolution and evolution of protein function, in particular, is a long-standing topic of keen interest in both experimental and theoretical aspects [1-3]. Recent advances in genomics and proteomics provided a wealth of sequences and structures, making it possible to unravel intricate evolutionary connections in the realm of protein function. Specifically, it became feasible to follow in detail convergence and divergence of protein function in case of speciation and adaptation [4,5], switching between natural and latent enzymatic activities [6], evolution of promiscuous functions [7], and recombination of functional domains into proteins with new functions [8]. There is still, however, an enigmatic question about the very emergence of the first enzymatic domains from primordial functional peptides. The ultimate goal would be to draw a picture of the emergence of functional domains/folds, their fate upon formation of proteomes and involvement into adaptation and speciation. First, it should be understood how protein structure started from combining the primitive peptides/proteins with elementary functions into folds with complex enzymatic activities. Then, the fusion and recombination of these folds into multidomain structures and protein complexes should be explored [9]. Further, the reutilization of already existing structures and the invention of new domains/folds with unique functions should be analyzed.

In order to dig as deep as to the emergence of the first enzymatic domains/folds, one has to hypothesize short peptides that preceded enzymes in the protein-RNA world. Existence of conserved functional motifs [10-12] in a big number of protein superfamilies suggests that they originated from ancestral peptides. Though severely changed, structural and functional “signatures” of these ancestors could survive in contemporary functional motifs. The first task, therefore, would be to define the unit of protein function and to use this definition for decomposing contemporary enzymatic functions into sets of elementary ones. Structurally, it has been shown that *closed loops* (or polypeptide chain returns) with a characteristic size 25 – 30 residues can be a common basic structural element of all globular proteins [13-18]. This element is apparently a consequence of the polymer nature of the polypeptide chains. Closed loops in modern proteins are also presumed to be units of protein domains [19], playing an important role in co-translational protein folding [20-22]. Functionally, the notion of *elementary functional loops* (EFLs), closed loops possessing the residues important for binding, activation, and catalysis has been introduced [10,23,24]. The EFLs are presumably descendants of primordial ring-like functional peptides of the protein-RNA world, which can be reconstructed in the form of sequence profiles with specific functional signature(s) and structure(s) of the closed loop (polypeptide chain return) [11]. The same elementary function can serve as a unit of different enzymes, forming their biochemical functions in combinations with other EFLs. As a result, descendants of a particular prototype can be found in unrelated folds and functions. Therefore, evolutionary connections unraveled by prototypes and EFLs go beyond homology on the functional superfamily level, illuminating the very process of building functional domains from the elementary units [10,11].

We use here the archaeal superkingdom as a model system for exploring the emergence and molecular evolution of the protein function. Archaea is an ancient superkingdom and has a compact structure with a clear division into four phyla: Crenarchaeota, Euryarchaeota, Korarchaeota, Nanoarchaeota. Archaeal species thrive in different extreme environments, such as high temperature and salinity, and most of them are anaerobes. All the above allows to explore the emergence of new functions in the process of speciation as well as in response to demands of the environment. One can trace, for example, evolutionary relations between the most common and ancient functions and those that emerged later. Earlier introduced notions of the archaeal “core” and the “shell” and the Last Archaeal Common Ancestor (LACA) [25] provide an excellent background for this analysis. The database of the archaeal Clusters of Orthologous Groups of proteins (arCOGs) gives the basic functional annotation for each cluster as well as its distribution across the archaeal lineages [26].

Some archaeal species possess unique enzymatic functions and even unique metabolic pathways. For example, methanogenesis [27] is a unique pathway found only in the archaeal divisions *MethanobacterialesMethanococcalesMethanomicrobiales* and *Methanosarcina* belonging to *Euryarchaeota* kingdom. Methanogens reduce carbon-containing compounds, such as CO_2_, formate, and acetate to methane in anaerobic conditions [27]. Overall, the carbon is subsequently transferred between three carbon-carriers: methanofuran (MF), tetrahydromethanopterin (H4MPT), and coenzyme M (CoM-SH) via seven major enzymatic steps of the methanogenesis pathway. These steps are very similar between all kinds of methanogens [27], and the main methanogenic enzymes are oxidoreductases and transferases [28]. One-carbon metabolism is considered to be one of the most ancient ones, and presumably of a prebiotic origin [29]. At the same time, genomic and geological evidences suggest that methanogenesis pathway evolved at rather late stages of archaeal evolution (2.8 billion years ago). There are in total more than 200 genes required for methane formation [30]. The majority of the proteins coded by these genes are involved into various coenzyme and cofactor biosynthesis, synthesis of prosthetic groups and ion transport. Despite the diversity of enzymes involved in the methanogenesis, they all evolved from the one ancestral set of enzymes [31]. We analyze two enzymes of the methanogenic pathways, identify structural folds and elementary functional loops, and discuss the plausible scenario of their emergence.

## Results and discussion

### Fold usage in the archaeal proteomes

**Figure 1:**
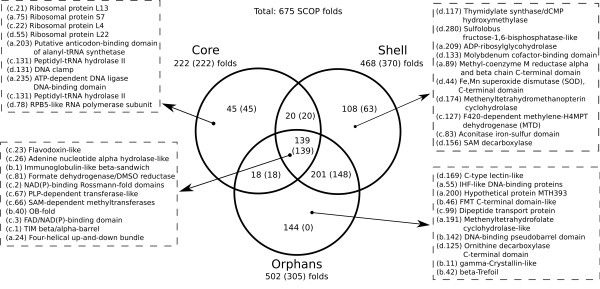
**The structural census of the archaeal COGs.** Venn diagram that shows the intersections of the sets of SCOP folds corresponding to the core (222/222 found in LACA), shell (468/370), and orphan (502/305) groups of arCOGs. For core only (45), shell only (108), orphans only (144), as well for the omnipresent set (139, the intersection in the middle of the diagram) ten folds are exemplified by their SCOP IDs and fold names. The complete sets of folds are given in Additional File 1.

Our goal here is to delineate evolutionary relationships between protein functions of the archaeal superkingdom via elementary functions comprising different enzymes. We analyze archaeal Clusters of Orthologous Groups (arCOGs) representing the whole proteins, protein complexes and their subunits, and identify the functions of their individual domains. The arCOGs are classified, according to their distribution across species, into the core (most common arCOGs, present in almost all archaeal species), shell (abundant, existing in more than 10 species), and orphans [26]. ArCOGs correspond to chains or subunits of complete proteins, which either can be fully functional by themselves or should be assembled into oligomeric protein complexes. Each arCOG, in turn, can be composed of several functional domains incorporated in a protein chain. It makes the functional domain an indispensable unit, which directly links enzymatic functions of modern proteins to primordial functional peptides. The reconstruction of evolutionary relations between protein functions should start, therefore, from establishing links between the functional domains comprising multidomain proteins and protein complexes. First, we detect SCOP folds [32] in the arCOG protein sequences. The 675 folds found in Archaea are distributed unevenly between the core, shell, and orphan arCOGs (shown in a Venn diagram in Figure 1). The core-only, shell-only, and orphan-only folds are exemplified with ten folds (complete lists of folds are available in Additional File 1). We also counted the number of folds (shown in parentheses) predicted to be in the Last Archaeal Common Ancestor (LACA) [26]. The diagram indicates the evolutionary dynamics in archaeal function and corresponding fold usage. Overall, there are two major options in the evolutionary fate of the functional domains/folds. Some of the specific folds are found only in the archaeal core (45), e.g. ribosomal protein folds, DNA and RNA binding folds (Figure 1). They were exclusively used for one or a few very common functions in the core and were not reused in other enzymes in the shell. These folds are present in all the archaeal lineages, which indicates their ancient and basic nature. On the other hand, there are many ancient core folds (139), such as Rossmann fold, FAD/NAD(P)-binding fold, and TIM β/α-barrel fold, which were reused in new (shell and orphan) functions (Figure 1, intersection of three circles). Every core fold is present in LACA (222 of 222), while the orphan-only folds (144) are not present in LACA at all. The difference between the core and orphan folds apparently indicates that most of the orphan and some of the shell folds represent the functional domains designed *de novo*. Among these new domains (Figure 1) there are, for example, unique folds of the methanogenic enzymes: Methenyltetrahydrofolate cyclohydrolase-like fold (a.191), F420-dependent methylene-H4MPT dehydrogenase fold (c.127), Methenyl-H4MPT cyclohydrolase (d.174), Methyl-coenzyme M reductase alpha and beta chain C-term fold (a.89).

### Metanogenesis pathway

We analyze proteins involved into methanogenesis pathway by using the set of profiles of elementary functional loops (EFLs) obtained for the whole archaeal superkingdom (the complete list of profiles is provided in Additional File 2). Elementary functional loops are represented by the sequence profiles in the form of 30-residue long position-specific scoring matrices (PSSMs). Additional File 3: Figure S1 shows the methanogenesis-related arCOGs and their connections to the non-methanogenic ones via profiles of EFLs. Since arCOGs are in many cases multidomain proteins or protein complexes (e.g. methanogenic enzymes formyl-MF dehydrogenase Fmd/Fwd, H4MPT S-methyltransferase Mtr, and methyl-CoM reductase Mcr), we split them into individual domains and consider functions and evolutionary connections of each domain separately (Additional File 3: Figure S2). There are novel folds, such as the folds of methenyl-H4MPT cyclohydrolase (Mch, 3^rd^ step) and N(5)-N(10)-methenyl-H4MPT dehydrogenase ( Mtd, 4^th^ step in the pathway) enzymes, which emerged in response to demand for new/specific function. Highly designable folds, such as β/α-barrel, Rossmann fold, and ferredoxin are abundant in the methanogenic enzymes. For example, β/α-barrel fold is present in Fmd/Fwd subunit A (catalyzing the 1^st^ step in the pathway) and Mer (5^th^ step); Ferredoxin fold – in Ftr (2^nd^ step) and in the other [Fe-S] cluster-containing enzymes, such as McrA and Fmd; Rossmann fold – in the Hmd enzyme (4^th^ step). There are also several rare coenzymes and cofactors working almost exclusively in methanogenesis, such as molybdopterin/tungsteen-pterin (MPT), coenzyme B (CoB-SH), coenzyme F_420_, and corrinoid cofactor F_430_. Despite their unique chemistry, some of these cofactors belong to wide groups of structurally similar chemical compounds. They may invoke therefore the similar chemistry of the recognition and binding, resulting in common elementary functional loops. In this case, the corresponding EFLs can be reused as building blocks of the new folds and biochemical functions. There are also cases where several folds fuse and make up multidomain enzymes, e.g. the unique two-domain structure of Mch. Some folds can assemble into protein complexes, such as the homoxehamer of the above-mentioned enzyme.

We consider here protein function starting from the level of elementary functional loops to functional domains and their combinations in mutidomain proteins and complexes. Below we analyze enzymes catalyzing the first (Fwd) and the last (Mcr) steps of the methanogenic pathway, and heterosulfide reductase (Hdr) enzyme linking these steps by reducing cofactors involved into them. For the details on these enzymes, see description in Additional File 3. The goal of this part is to show different ways of the function emergence and evolution such as reutilization of the abundant folds in forming specific methanogenic enzymes, the role of elementary functional loops as building blocks of the new enzymes, and formation of the protein complexes.

### Different EFLs working in domains of formyl-methanofuran dehydrogenase (Fwd)

**Figure 2:**
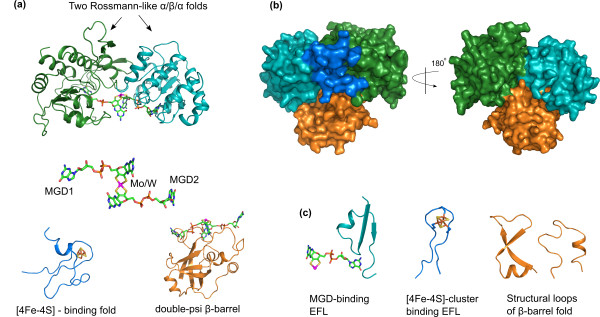
**Homology model of formyl-methanofuran dehydrogenase enzyme (Fwd).** The structures of Fwd subunits B and D from *M. jannashii* were modeled using structural templates from *E.coli* and *A. fulgidus*. (**a**) FwdB consists of three domains. Two of them have Rossmann-like α/β/α folds (green and cyan). The B and D subunits together bind two molecules of molybdenum cofactor dinucleotide MGD [1,2] shown in sticks representation. The third domain binds a [4Fe-4S] cluster (blue). The subunit D with a β-barrel fold (orange) is also involved in (di)MGD cofactor binding. (**b**) Surface representation of FwdBD protein complex model with the individual domains colored as in chart a, rotated 180 degrees around the y-axis. (**c**) Structures of the elementary functional loops (left-to-right): MGD cofactor binding EFL (cyan) from one of the Rossmann-like domains is shown together with one MGD molecule; iron-sulfur cluster-binding EFL (blue) from the ferredoxin-like fold together with the [4Fe-4S] cluster; two psi-loops (orange) constituting the core of the double-psi β-barrel fold are also involved in MGD binding.

Figure 2 shows the homology model of *Methanococcus jannaschii* formyl-methanofuran dehydrogenase enzyme (Fwd, subunits B and D) built using the templates formate dehydrogenase FdhH from *E. coli* and FwdD from *A. fulgidus*. In SCOP database [32] the whole subunit B is classified as one fold (c.81, Formate dehydrogenase/DMSO reductase) and represents the domain as an evolutionary/functional, rather than structural unit. The subunit B, however, clearly consists of several structural domains. Two of these domains (green and cyan, Figure 2a,b) have Rossman-like α/β/α (di)nucleotide-binding folds (similar to c.2, c.3, and c.23 core folds in Figure 1). These are highly designable ancient folds, which were (re)utilized in many functions starting from the core ones (see Figure 1, where c.81 is one of the core folds). In Fwd these two domains work together and bind two molecules of the molybdopterin(tungstopterin) guanine dinucleotide cofactor (MGD, shown in backbone representation in Figure 2a) connected via a molybdenum atom (tungsten in Fwd). The elementary functional loop 515 with the signature -Rx [TS] × [TS]AxxADx(6)PG[TS]D- is likely responsible for the MGD-binding (Figure 2c, cyan loop). The third domain (blue) is a common [4Fe-4S]-binding fold. Iron-sulfur cluster has an important role in Fwd catalytic function, providing an intermediate step in the redox reaction as an electron carrier. The EFL found by profile 500 with the characteristic cysteine-rich signature -CxxCxxCxxxCP- of the [4Fe-4S] cluster binding (see also [11]) is shown in Figure 2c (blue). There is another domain with quite unusual double psi β-barrel fold (orange). It contains a structural core formed by two elements with specific turn angles (Figure 2c, orange loops). It has been previously shown that the enzymes with a double psi β-barrel fold have their functional sites around this core [33]. In Fwd the domain with a double psi β-barrel fold is a separate subunit, whereas in FdhH it is part of the active site that binds the MGD cofactor together with the Rossmann-like folds. This additional domain presumably contributes to the specificity of cofactor binding via hydrogen bonds between the psi loop and the MGD [33,34].

### Reutilization of folds and EFLs in cofactor F_430_ binding

**Figure 3:**
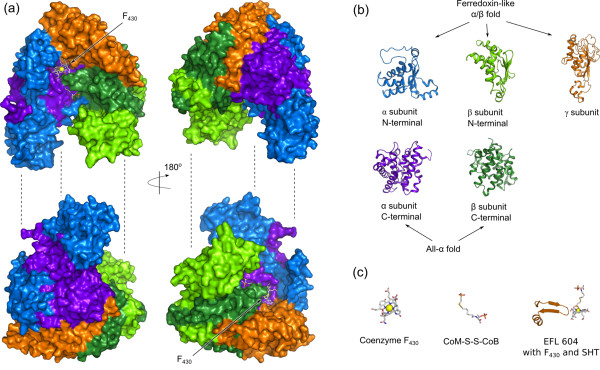
**The subunits of methyl-coenzyme M reductase (Mcr).** (**a**) The structure of Mcr (PDB ID: 1HBM) consists of two alpha, two beta, and two gamma subunits. The structure is split into two parts, each consisting of one set of subunits for clarity. The subunit alpha consists of two domains: the N-terminal domain (marine) and the C-terminal domain (dark-violet). The subunit beta also consists of two domains: the N-terminal (light-green) and the C-terminal (dark-green). The subunit gamma is shown in orange color. Two molecules of the cofactor F_430_ are shown together with the substrate heterosulfide (positions indicated by arrows). The pair of split structures on the right is rotated 180 degrees around the y-axis. (**b**) The structures of the individual domains of the Mcr subunits shown in ribbon representation. The colors are the same as in the chart a. N-terminal domains of subunits alpha and beta, and subunit gamma originate presumably from the common origin with the ferredoxin-like fold. C-terminals of subunits alpha and beta have all-α fold. (**c**) left-to-right: the structure of cofactor F_430_ in stick representation with the nickel atom shown as a yellow sphere; heterosulfide CoM-S-S-CoB in oxidized form; elementary functional loop with cofactor F_430_ (from the subunit gamma) corresponding to the profile 604.

Figure 3a shows Methyl-coenzyme M reductase (Mcr), which is a hexamer consisting of two alpha, two beta, and two gamma subunits coordinating two F_430_ cofactor molecules [35]. This protein represents an interesting example of fold reutilization. All Mcr subunits apparently originate from the two basic folds: a ferredoxin-like fold and an all-α fold. The structure is divided in the Figure 3 into two halves for clarity, each consisting of one alpha (blue), one beta (green), and one gamma (orange) subunit. Figure 3b shows that alpha and beta subunits consist of two structural domains. The N-terminal domains originated from a ferredoxin-like fold (marine and light-green), and the C-terminal domains have all-α fold (dark-violet and dark-green). The subunit gamma consisting of one structural domain (orange) is also derived from a ferredoxin-like fold. The elementary functional loops interacting with F_430_ cofactors have distinctive signatures. The EFL with the active Tyr367 (in PDB 1hbm) coordinating the nickel atom in F_430_ and directly interacting with CoM-CoB ligands has a glycine-rich signature -YGGGGPG-. Another EFL represented by profile 604 with the signature -RGxDxG [TS]LSGRQxxExRExDxExxxK- interacts with F_430_ in Mcr, and it is directly involved in the methyl group transfer. Profile 400 with the generalized signature -GxDxGxxG- appears to be the more general description of the same elementary function as represented by the profile 604. According to PDBeMotif tool, the signatures of both profiles are related to binding of nucleotides and dinucleotides (e.g. in FAD, NAD, and ADP), binding pyridoxal-5'-phosphate (PLP), and Coenzyme M (CoM).

### Reutilization of the same elementary function in different domains of heterosulfide reductase (Hdr)

Hdr enzyme is a protein complex composed, in the most general case, of three subunits: HdrA, HdrB, and HdrC. In *Methanothermobacter* HdrABC forms a complex with another enzyme [NiFe]-hydrogenase Mvh [36] and acts as an electron acceptor. HdrABC uses the electrons obtained from Mvh to reduce ferredoxin and heterosulfide. The structure of heterosulfide reductase (Hdr) has not been resolved yet, however it is possible to explore the functions of its subunits using sequence profiles of the elementary functional loops. The subunit A of the Hdr contains several ferredoxin reductase-type FAD-binding motifs. In the -[RH]x[FY][TS]- motif the R/H form hydrogen bonds to the phosphate oxygen atom [37]. Additionally, HdrA contains four motifs for binding [4Fe-4S] clusters with the common signature -CxxCxxCxxxC- represented by profile 500. The C subunit of Hdr contains two [4Fe-4S] cluster-binding EFLs with the same signature, as in subunit A. HdrB is the catalytic domain, which uses another [4Fe-4S] cluster (bound with a different cysteine-rich signature) and also contains several redox-active cysteine residues.

### Evolutionary relations between superfamilies of archaeal functions

The task of this part of the paper is to find evolutionary connections going beyond homology in enzyme superfamilies. Three main reasons for the evolutionary connections between the arCOGs and their functions can be named: (i) domain fusion and recombination, as some arCOGs are multidomain proteins and/or protein complexes; (ii) proteins in connected arCOGs are distant homologs diverged from the same ancestral domain/fold; (iii) common elementary functions are present in different non-homologous arCOGs. The latter describes, for instance, common steps in the biochemical transformations or the binding of chemically similar substrates or cofactors. The connections between proteins sharing elementary functions can originate from the primordial evolution, hence they are not restricted within a (super) family or fold. We consider here functional domains and links between them provided by the elementary functional loops and their prototypes. Functions of individual domains rather than those of the whole proteins are analyzed, since the first enzymatic domains were presumably formed from the simple primordial peptides with elementary functions [9]. We start from the arCOGs in the archaeal core (preferably single-domain arCOGs), as they represent the most common protein functions (Figure 1). Then we identify the elementary functional loops, which served as building blocks of these domains and provided key steps of their biochemical functions. Below we show how EFLs unravel intricate connections between protein superfamilies with different biochemical functions.

**Figure 4:**
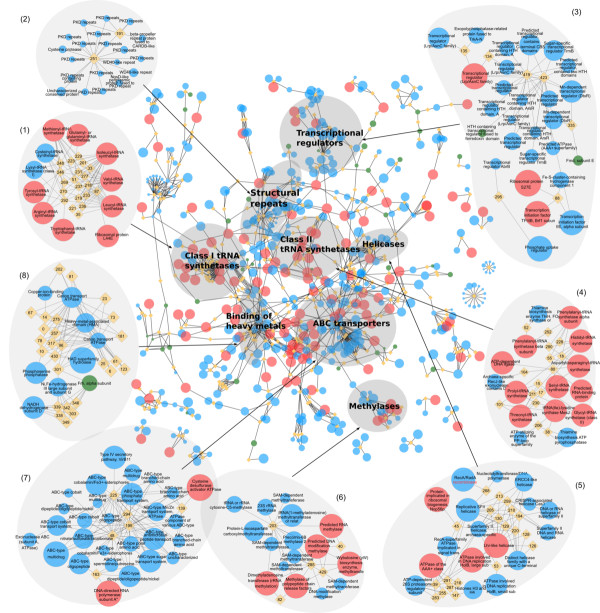
**Connections between archaeal functions via the sequence profiles of elementary functional loops.** (**a**) Clusters of arCOGs. Circular nodes represent arCOGs. The size of the node represents the number of lineages where arCOG is present. The core arCOGs are colored red, shell - blue, and arCOGs involved in methanogenesis pathway - green. Sequence profiles of the elementary functional loops are represented as orange diamonds in the graph. The edges between the diamonds and the circles represent profile-arCOG matches. The selected clusters of arCOGs are labeled according to their common biological functions: (**1**) class I aminoacyl tRNA synthetases, (**2**) structural repeats, (**3**) transcription regulators, (**4**) class II tRNA synthetases, (**5**) helicases, (**6**) methylases, (**7**) ABC transporters, (**8**) binding of heavy metals.

**Figure 5:**
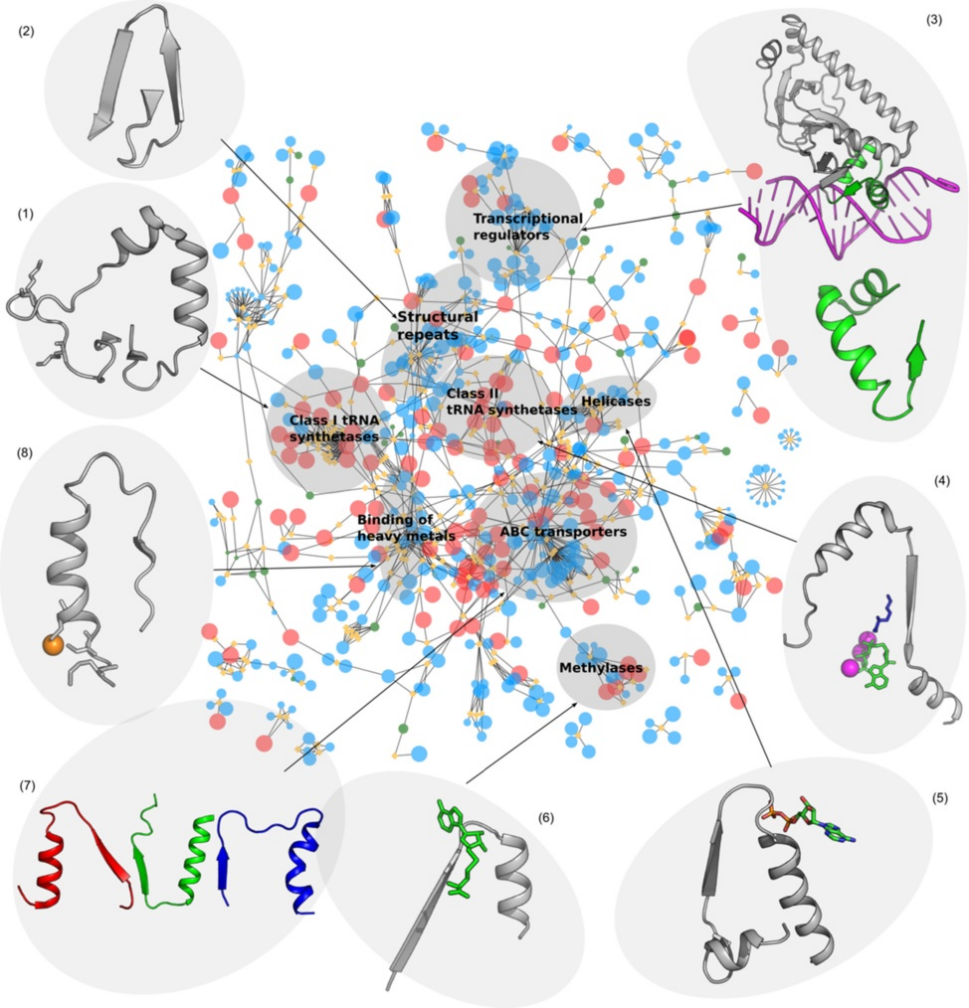
**Examples of the structures of key elementary functional loops in the clusters of arCOGs.** (Cluster 1) the profile 292, signature -GxKMSKSxG- (PDB ID: 1FFY, chain A, residues 588–633) two lysines are shown as sticks; (cluster 2) the profile 251 -WxFGDGx(11)Y- (PDB ID: 1B4R, chain A: 38–58) structural repeat comprising Ig-like fold of PKD; (cluster 3) the profile 423 (IAx(9)Vx(6)LxxxGxV) Catabolite gene activator protein (PDB ID: 1RUN, chain A) in complex with DNA; (cluster 4) the profile 280 with the signature -Px(2)GxGxGxxRL- represents one of the three characteristic motifs in aminoacyl tRNA synthetases Class II core domains. The EFL corresponding to the profile 280 is shown for the structure PDB ID: 1E24, chain A, which is Lysyl-tRNA synthetase together with Lysine (blue), ATP (green), and Mn^2+^ ions (magenta); (cluster 5) “Helicases”: the profile 45 -Lx(3)Px(3)GKTLxAExA- (PDB ID: 2DR3 chain A: 9–51) recA family protein with ADP bound; (cluster 6) the profile 429 (VxGxDx(8)A) PDB ID: 1BC5 chain A chemotaxis receptor methyltransferase with S-adenosyl-L-homocysteine (SAM-analogue); (cluster 7) the profile 225 (Gx(6)GxxGxGKTT), the profile 195 (LSGGxxQRxxxAxxLxxxPxxxxxDEPxxxLD), profile 294 (GxxxQx(12)N); (cluster 8) the profile 14, signature -GMxCxxCxxxVx(8)GV- (PDB ID: 1KOV chain A:10–37). The copper ion is coordinated by Met and two Cys (shown in sticks representation) in the elementary functional loop.

Elementary functional loops are represented by the sequence profiles in the form of 30-residue long position-specific scoring matrices (PSSMs). In some cases, a profile represents enzymes belonging to different families and even superfamilies. The complete set of profiles with related elementary functions represents the prototype with the signature of the most basic and common elementary function. This prototype describes the primordial ancestor of the related EFLs. Using the procedure described in Materials and Methods, we derived 525 sequence profiles from the archaeal proteomes (complete list is given in Additional File 4) and matched them to the sequences of the arCOGs. We found that some profiles match to several arCOGs. Figures 4 and 5 show connections between the core arCOGs (represented as red nodes) via profiles of the elementary functional loops (orange diamonds). Ancient elementary functions of the core are also frequently found in the arCOGs present in the “almost-core” arCOGs (between 25 and 38 species) shown by the large blue nodes in Figures 4 and 5. The size of the node represents the number of archaeal species possessing the arCOG. In order to exemplify evolutionary connections revealed by the elementary functions, we identified arCOGs clustered around particular sequence profiles with elementary functions typical for the cluster (shown in Figure 4). We name these clusters according to the prevailing enzymatic function of the arCOGs and consider connections between them.

### Aminoacyl tRNA synthetases

Two clusters of arCOGs representing aminoacyl tRNA synthetases (aaRS) are determined based on the sets of the corresponding profiles. The catalytic domains of aaRS are highly conserved, and there are several distinct signatures connecting synthetases of the same class. For example, class I aaRS (see Figure 4, cluster 1) is characterized by the profile 292 with the signature -GxKMSKSxG-. The elementary functional loop containing this characteristic signature is a part of the active site in aaRS class I, where the second lysine stabilizes the aminoacyl-adenylate. Figure 5 shows the structure of the corresponding EFL. The elementary function of this EFL is the adenine moiety recognition and binding via a hydrogen bond [38]. Additionally, aaRS of the class I are also connected by the elementary functions of heavy metal and ion transport possessed by the profile 177 with the signature -GDGxxD-. This functional signature describes interactions with Ca^2+^ and Mg^2+^ ions (according to PDBeMotif database of protein-ligand interactions [39]). Class II aaRS is also interconnected by several typical profiles (Figure 4, cluster 4). For example, the profile 280 has a characteristic glycine-rich signature -Px(2)GxGxGxxRL-, similar to the nucleotide binding signatures. Figure 5 contains the example of the EFL corresponding to the profile 280, where an elementary functional loop from a Lysil-tRNA synthetase is shown together with substrates Lysine and ATP.

### Structural repeats

It is known that structural repeats are typical for many proteins, including β-propellers, PKD domains, WD40 domains, and cell surface proteins [40-42]. Although the corresponding arCOGs are not related functionally, profiles 251 (−WxFGDGx(11)Y-) and 191 (−PxIxGx(2)IVWxD-) represent repeating motifs which serve as conserved structural building blocks (Figure 4, cluster 2). Figure 5 illustrates the example of a typical structural repeat comprising Immunoglobuline-like fold of PKD domain with the signature of the profile 251.

#### Transcriptional regulators

Transcriptional regulators belonging to different families (DtxR, HTH Helix-turn-helix, TrmB, cBS, Lrp/AsnC transcription initiation factors) share several functional signatures (Figure 4a, cluster 3). One of them is exemplified by the profile 423 with the signature -IAx(9)Vx(6)LxxxGxV-. The “HTH regulator fused to ferredoxin domain” (green circle) is the example of the connection as a result of domain fusion. In this case the arCOGs is a multidomain protein. Figure 5 shows the Catabolite gene activator protein (CAP), which is a complex of the transcription factor with DNA. The EFL corresponding to the profile 423 (green) provides the interface between the protein and DNA. Presumably, this elementary function of DNA-binding is also used by the other arCOGs where matches of the profile 423 were found.

### Helicases

Helicases, ATPases involved in replication, replicative SHII helicases, and recombinases have common elementary functional loops responsible for the interactions with nucleic acids (Figure 4, cluster 5). For instance, the profile 45 has a typical signature of helicases − Lx(3)Px(3)GKTLxAExA- [43,44]. This profile connects several protein superfamilies: RecA-superfamily ATPase implicated in signal transduction, protein implicated in ribosomal biogenesis, superfamily II helicase, and replicative SFII helicase superfamily. Figure 5 shows an example of the elementary functional loop representing the profile 45 in RecA-superfamily, where its function is the ADP binding.

#### Methylases and methyltransferases

Methylases/methyltransferases are involved in the addition/transfer of methyl chemical group via nucleophilic and radical mechanisms [45]. The group of methylases (Figure 4, cluster 6) has several characteristic signatures, for instance profiles 203 (−VLDxGxGxGx(6)A-) and 429 (−VxGxDx(8)A-). Figure 5 shows an example of the EFL representing the profile 429 in chemotaxis receptor methyltransferase with a bound S-adenosylmethionine (SAM) analog. It indicates that the same elementary function is shared between the SAM-dependent methyltransferases, different RNA methylases, including tRNA and rRNA methylases, and biosynthesis enzymes with the methyltransferase activity.

#### ABC transporters

The ATP binding cassette is a common component of the ABC transporters cluster (Figure 4, cluster 7). The cluster includes ATPase component, transport systems for metal ions, amino acids, drugs, and small peptides. The ATP binding cassette consists of several highly conserved [46] consecutive functional signatures. We found profiles corresponding to the major functional loops in the ABC transporters. The profile 225 (−Gx(6)GxxGxGKT-) corresponds to the Walker A motif (also called P-loop), which interacts with the phosphate groups of the nucleotide in the ATP. The profile 195 (−LSGGxxQRxxxAxxLxxxPx(5)DEPxxxLD-) contains several signatures. First, it includes the Walker B motif, which coordinates the Mg^2+^ ion and provides a water molecule polarization. The profile 195 also contains a typical signature of all nucleotide hydrolases (−LSGG-), acting as a γ -phosphate sensor. Additionally, this profile includes a D-loop signature with conserved (−LD-) residues. The profile 294 (−GxxxQx(12)N-) represents the so-called Q-loop with a highly conserved glutamine, providing a nucleophilic attack of the γ-phosphate in the ATP [47]. Figure 5 shows the structure of MalK – an ATPase subunit of the sugar ABC transporter in the archaeaon *Thermococcus litoralis* and is a representative example of the “ABC transporters” cluster [48]. It contains three major elementary functional loops, representing profiles characteristic for the ABC transporters: 225, 195, and 294. Finally, elementary functions working in ABC transporters are also present in other biochemical functions where ATP binding is part of the reaction. For example, elementary functional loops of the profiles 225 and 195 work in the Cysteine desulfurase activator ATPase.

#### Metal binding

Heavy metals, such as Mo, W, Co, Mg, and Cu are widely used in enzymes in various biochemical and cellular processes as cofactors in the catalysis, activators, and electron donors/acceptors in redox reactions [49]. Cluster 8 in the Figure 4 shows several profiles with the elementary function of a metal binding. For instance, the profile 14 with the signature − GMxCx(2)Cx(3)Vx(8)GV- characterizes elementary function of the copper binding (example of EFL structure is shown in Figure 5). Highly conserved cysteines and a methionine residue constitute the functional signature of this profile. In the graph in Figure 4 the profile 14 connects the Copper-ion-binding protein superfamily with the Cation-transport ATPase superfamily. Both superfamilies share the elementary function of the copper binding. Recently we showed that the profile 14 is a derivative of the ancient prototype with the generic signature -CxxC- and the general elementary function of the metal and metal-containing cofactor binding [11]. Another example, the profile 10 with the signature − Vx(3)GDGxNDAxALx(2)Ax(2)GxA- binds various ions and inorganic compounds (AlF_4_, BeF_4_, K^+^, Ca^2+^, MgF_4_, Mg^2+^, and Na^+^, according to PDBeMotif [39]). In the arCOG graph (Figure 4a) the profile 10 connects several protein superfamilies: HAD superfamily hydrolase, Cation transport ATPase, and Heavy metal associated domain (HMA) superfamily.

Above examples of elementary functions include binding, activation, and elementary reactions, which presumably existed in the prebiotic RNA-protein world and served as basic units in the formation of the first enzymatic domains. The binding of metals with generalized -CxxC- and Aspartic-rich signatures (*e.g.* profiles 10, 14, and 177) and the phosphate group binding characterized by glycine-rich signatures (*e.g.* -GxxGxG-) are the examples of abundant and presumably ancient elementary functions.

## Conclusions

Contemporary proteins are sophisticated molecular machines built of hundreds or thousands amino acid residues. Structurally, they consist of the independent and compact domain(s) formed by the continuous polypeptide chain or several protein chains interacting and forming a protein complex. This work attempts to draw a picture of protein evolution starting from the prebiotic evolution of protein-like molecules with elementary functions and spanning into the contemporary evolution of protein structure and function.

We relied here on the concept of elementary functional loop (EFL) as a presumed basic unit of the protein function. We derived sequence profiles of EFLs using the set of complete proteomes from the archaeal superkingdom. Our analysis shows that in the earliest stages of protein evolution or even earlier in the prebiotic world, combinations of primitive peptides/proteins with elementary functions, such as nucleotide/phosphate (Figures 4 and 5, clusters 1, 4, 5, and 7) or metal cofactor (Figures 4 and 5, cluster 8) binding apparently formed the first enzymatic domains. The most designable folds (such as β/α-barrel and Rossmann fold) apparently served as scaffolds for biochemical functions of the first enzymatic domains. The enzymes with different folds can contain elementary functional loops that diverged from the ancestral peptides with particular functions. Therefore, functional relations between enzymatic domains could have been established already in the predomain evolution when the first functional folds have been formed. Figure 4 contains examples of common biochemical functions (represented by arCOGs) clustered around the key elementary functional loops. In many cases there is one or a few EFLs, which determine the clustering of enzymes.

We used methanogenesis pathway as a case study in order to show how enzymes with new functions can be formed from elementary functions and via reutilization of already existing functional domains. Methanogenesis is only observed in Archaea, moreover only in few lineages, and is characterized by several unique folds and unusual cofactors [27]. We considered enzymes catalyzing the first (Fwd) and the last (Mcr) steps of the methanogenesis in detail. The subunits FwdB and FwdD exemplify how binding of two molecules of molybdopterin dinucleotide cofactor (MGD) is achieved by the mutual work of two Rossmann-like (di)nucleotide binding folds fused together. These two folds (Figure 2, cyan and green) form the catalytic domain together with a [Fe-S] cluster-binding (Figure 2, blue) and a beta-barrel (Figure 2, orange) folds with elementary functions supporting the MGD binding. The Mcr enzyme catalyzing the last step in methanogenic pathway has several different subunits evolved from the two folds: a ferredoxin-like fold and an all-α fold, which are used in several copies for building of the enzyme. Mcr is also an example of utilizing metal- and nucleotide-binding signatures involved in interactions with a unique F_430_ cofactor. Using another enzyme Hdr, we show how very similar elementary functions of [Fe-S] cluster binding can be used in different combinations in order to build a complex enzyme with oxidoreductase activity.

To conclude, clear phylogenetic structure with four well-characterized phyla, a long evolutionary history bordering to the origin of life in the prebiotic world, and a diversity of colonized environments made Archaea an attractive subject for the studies of the evolution of protein function. We were able to analyze major ways of the emergence and evolution of the protein function and to show how to reconstruct evolutionary relations between different enzymes. The future task we foresee is two-fold: (i) to obtain a set of elementary functions, which would exhaustively describe chemical transformations existed in a prebiotic world; (ii) to determine the original set of enzymatic domains that formed from the above elementary functions and served as a seed in the evolution of the protein function.

## Methods

### Core and shell arCOGs

We used arCOG database comprising 41 archaeal proteomes [26]. The definition of the core differs slightly from the original one and includes arCOGs present at least in 39 species. We excluded from consideration *Nanoarchaeum equitans*, which is an obligatory symbiont and lacks a large number of core proteins due to its lifestyle. If we were to include *N. equitans* in the core, the number of core arCOGs would be only 79 instead of 166. We also missed *Termoproteus tenax*, because its genome was not publicly available at the time of the study. The shell (arCOGs present at least in 10 species), orphans (less than 10 species), and LACA (Last Ancestral Common Ancestor) groups of arCOGs are defined according to the database.

### Detecting domains in arCOGs

We used HMM library from Superfamily database [50] based on ASTRAL/SCOP release 1.75 [32,51] in order to detect SCOP folds in arCOGs [26]. A complete list of detected SCOP folds in the core, shell, and orphan arCOGs is provided in Additional File 1.

### Obtaining sequence profiles of elementary functional loops

We used 30-residue long segments from the sequences of arCOGs as origins for deriving profiles of elementary functional loops (EFLs). The origins were iteratively matched against 68 non-redundant (70% sequence identity) archaeal proteomes until they converged into sequence profiles. Afterwards, the profiles were clustered in order to remove any remaining redundancy. The procedures for converging and clustering profiles are described in detail elsewhere [10,11]. The computational pipeline yielded 525 sequence profiles with distinct functional signatures. We refer to the profiles by their serial numbers or by PROSITE-like patterns uniquely identifying their signatures. Additional File 2 contains logos of all the profiles. The list of archaeal proteomes is provided in Additional File 4.

### Connections between the arCOGs

Using profile-sequence search [10] we looked for the matches between the derived profiles and non-redundant arCOG sequences with the expected number of false hits less than one. In order to increase the robustness of profile-arCOG matches we excluded connections having less than 15 matches (five matches for methanogenic arCOGs). The resulting connections were visualized using Cytoscape 2.7.0 [52].

### Assigning the elementary function

Sequence profiles of elementary functional loops were used to find matches in CDD and SCOP domains with known structure [32,53]. For many protein families functionally important residues are known, and the role of the latter in binding [39], intermolecular interactions [54], and mechanism of catalysis [55] was used to assign the profiles their elementary functions.

### Methanogenic arCOGs

The methanogenic enzymes were identified by taking KEGG orthologous groups from methane metabolism pathway [56] and finding the corresponding COGs and arCOGs. The major enzymes of the methanogenic pathway and some common enzymes involved in co-factor biosynthesis are listed in Additional File 3.

### Homology modeling of formyl-methanofuran dehydrogenase (Fwd)

We modeled two subunits FwdB (UniProt AC: P61154) and FwdD (UniProt AC: Q58568) from *M. jannashii* based on two structural templates: formate dehydrogenase H (FdhH) from E.coli (PDB ID: 1fdo chain A) and FwdD from *Archaeoglobus fulgidus* (PDB ID: 2ki8 chain A). We used SWISS-MODEL server [57] with a fully automated modeling procedure. FwdB contains two α/β/α Rossmann-like folds and a small Fe-S cluster-binding domain, whereas FwdD is a beta-barrel fold. The template FdhH contains the domains homologous to both subunits FwdB and FwdD in one chain, therefore, we were able to model the FwdB-FwdD complex assuming that domain interactions are conserved. We assembled the complex by aligning *A. fulgidus*-based model of subunit FwdD to the corresponding beta-barrel domain of FdhH template (from *E.coli*).

## Competing interests

The authors declare that they have no competing interests.

## Authors’ contributions

AG carried out the computational experiments. AG and INB designed the study, analyzed the data, and drafted the manuscript. All authors read and approved the final manuscript.

## Supplementary Material

Additional file 1 Contains the complete lists of SCOP folds found in the core, shell, and orphans groups of arCOGs, as well as in the intersections between the groups. Click here for file

Additional file 2 Contains the logo representation of the sequence profiles of elementary functional loops. Click here for file

Additional file 3 **Contains description of enzymes involved in methanogenesis, the list of the corresponding arCOGs (Additional file**3**: Table S1), and the graphs of connections between methanogenic arCOGs (Additional file**3**: Figure S1) and the domains comprising them (Additional file**3**: Figure S2) [**[29]**,**[35]**,**[58-61]**].** Click here for file

Additional file 4 Contains the list of archaeal proteomes used to obtain sequence profiles. Click here for file
