# Supplementary material for: Exploring the evolution of protein function in Archaea
Source: BMC Evol Biol. 2012 May 30;12:75. doi: 10.1186/1471-2148-12-75 (PMC3458885; doi:10.1186/1471-2148-12-75)
Supplement: Additional file 2 — Contains the logo representation of the sequence profiles of elementary functional loops. [file 1471-2148-12-75-S2.pdf]

Exploring the evolution of protein function in Archaea

Alexander Goncearenco and Igor N. Berezovsky

Additional File 1

The list of 525 sequence profiles. Sequence logo were generated with Weblogo 3. The numbering is sparse. Profiles with serial numbers above 500 were not clustered in order to remove redundancy, therefore there can be similar logos in the list. The value K specified in brackets denotes the number of non-redundant archaeal sequences comprising the profile.

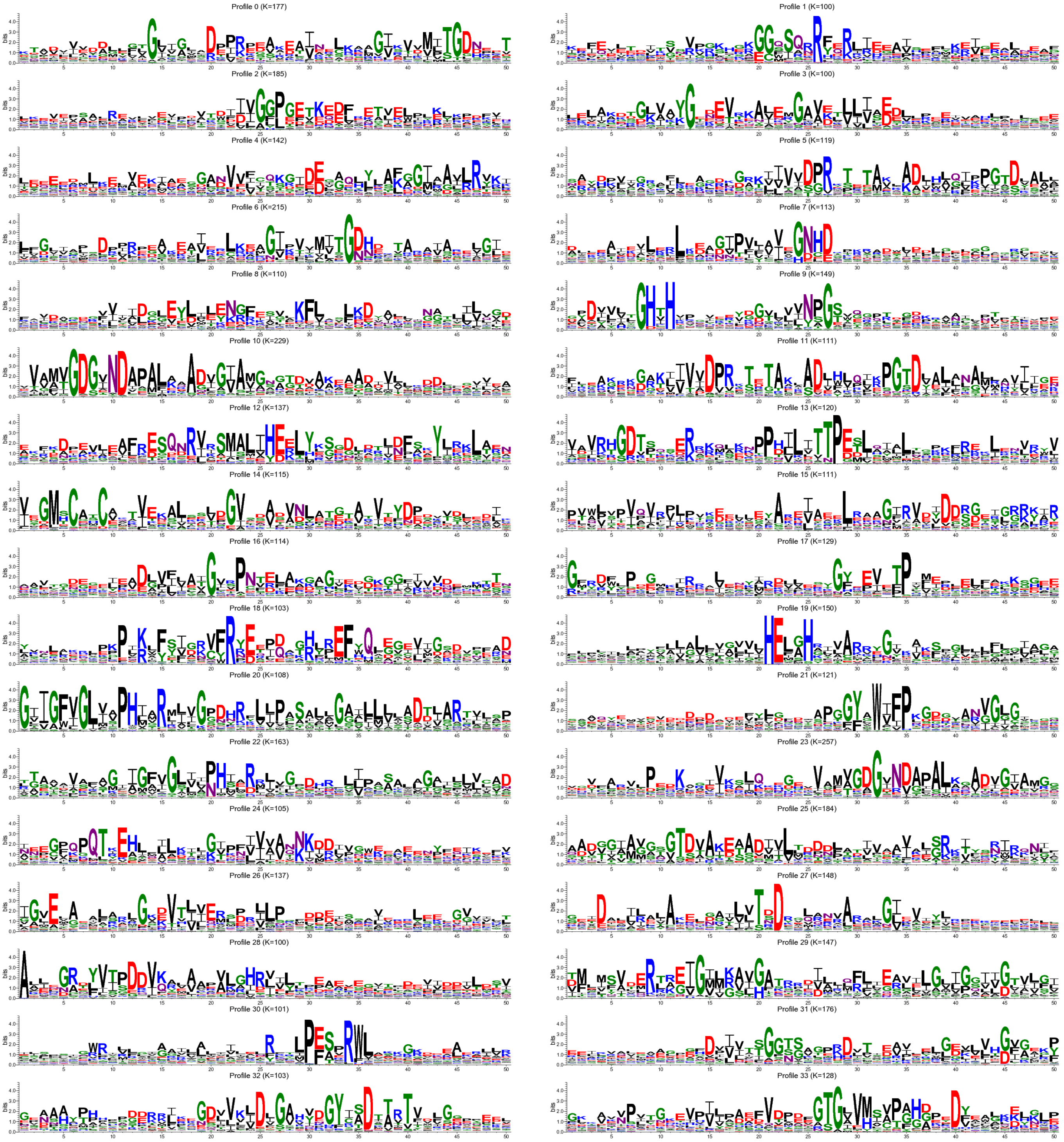

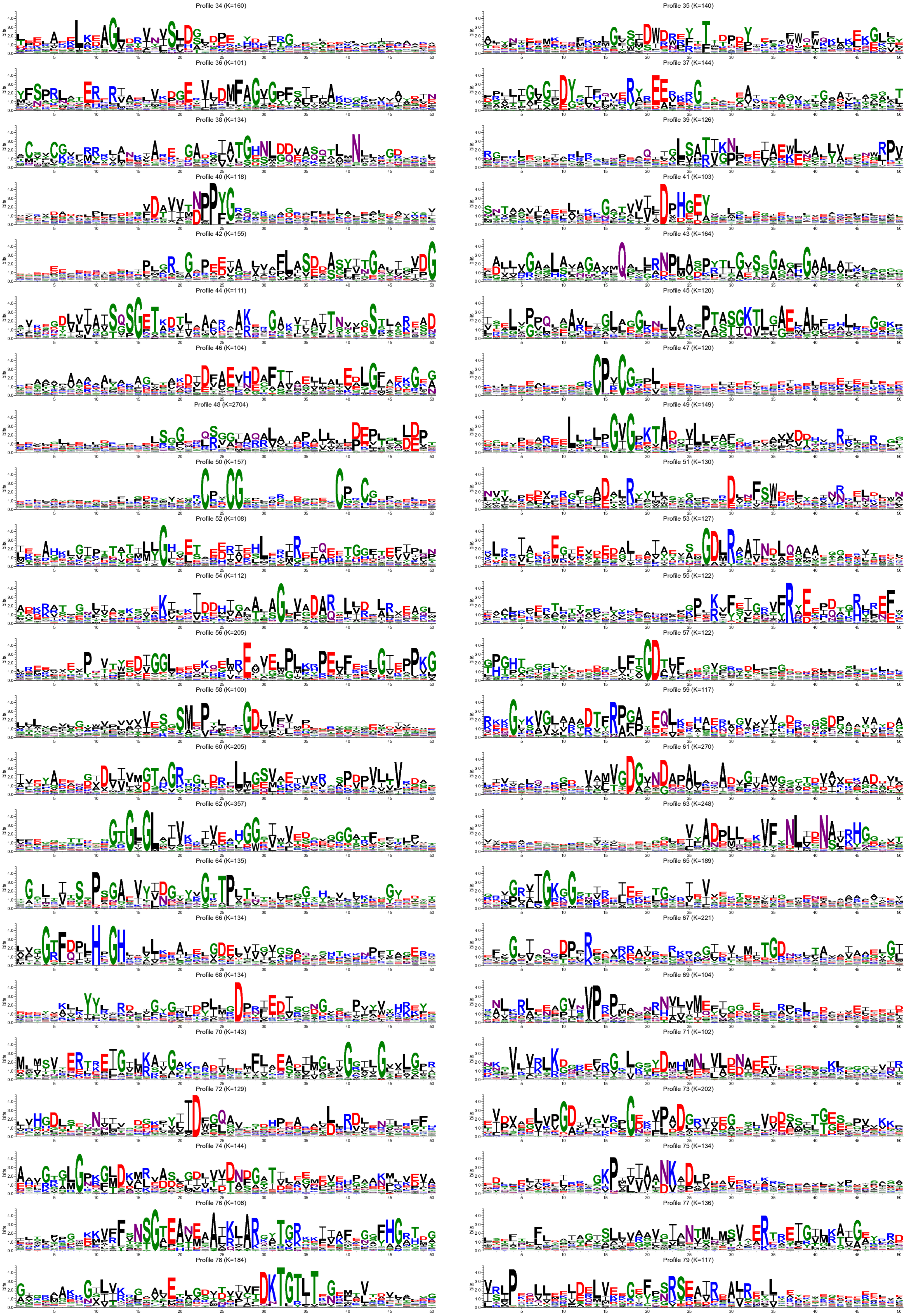

Profile 34 (K=160)
Profile 35 (K=140)
Profile 36 (K=101)
Profile 37 (K=144)
Profile 38 (K=134)
Profile 39 (K=126)
Profile 40 (K=118)
Profile 41 (K=103)
Profile 42 (K=155)
Profile 43 (K=164)
Profile 44 (K=111)
Profile 45 (K=120)
Profile 46 (K=104)
Profile 47 (K=120)
Profile 48 (K=2704)
Profile 49 (K=149)
Profile 50 (K=157)
Profile 51 (K=130)
Profile 52 (K=108)
Profile 53 (K=127)
Profile 54 (K=112)
Profile 55 (K=122)
Profile 56 (K=205)
Profile 57 (K=122)
Profile 58 (K=100)
Profile 59 (K=117)
Profile 60 (K=205)
Profile 61 (K=270)
Profile 62 (K=357)
Profile 63 (K=248)
Profile 64 (K=135)
Profile 65 (K=189)
Profile 66 (K=134)
Profile 67 (K=221)
Profile 68 (K=134)
Profile 69 (K=104)
Profile 70 (K=143)
Profile 71 (K=102)
Profile 72 (K=129)
Profile 73 (K=202)
Profile 74 (K=144)
Profile 75 (K=134)
Profile 76 (K=108)
Profile 77 (K=136)
Profile 78 (K=184)
Profile 79 (K=117)

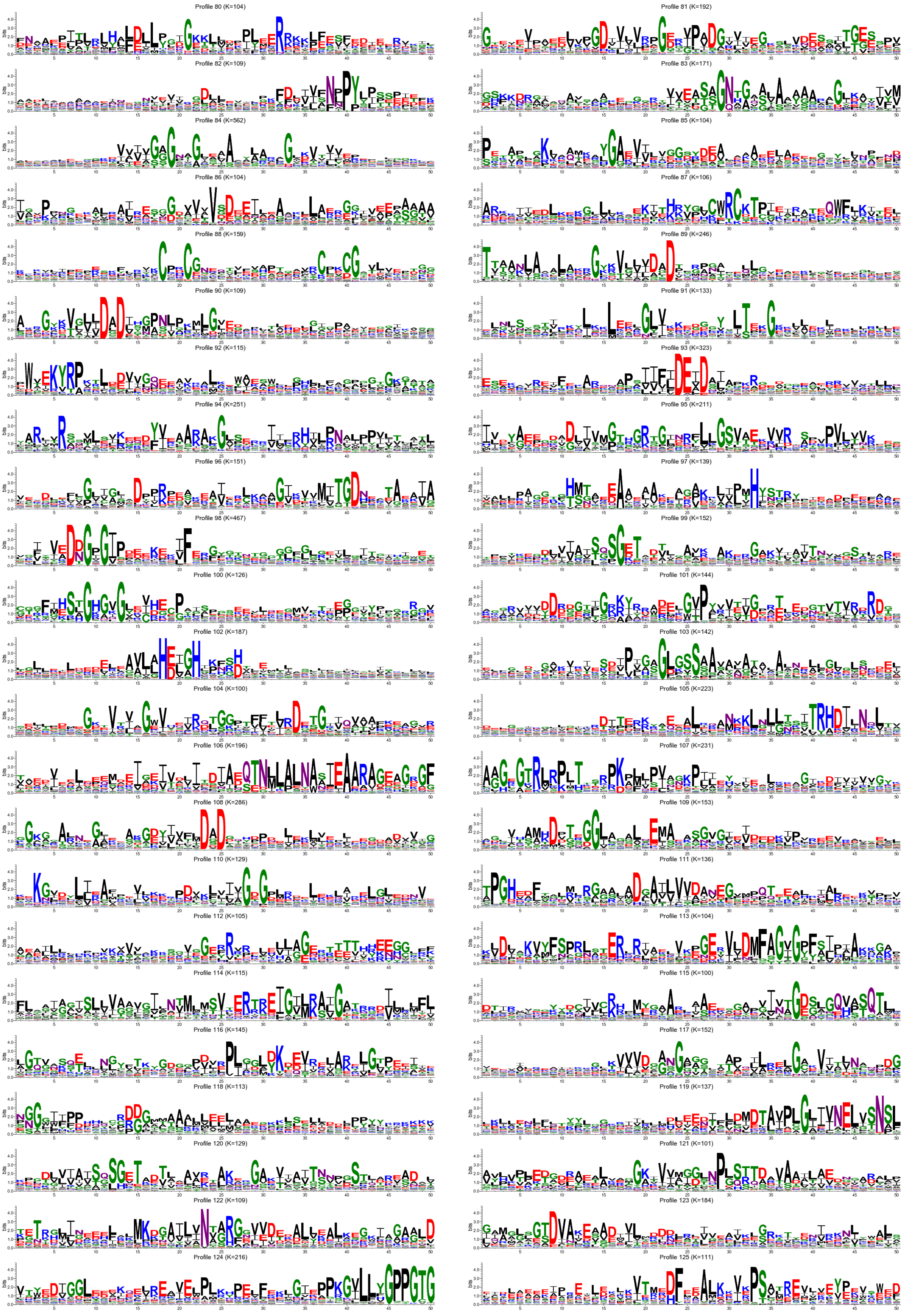
Profile 80 (K=104)
Profile 81 (K=192)
Profile 82 (K=109)
Profile 83 (K=171)
Profile 84 (K=562)
Profile 85 (K=104)
Profile 86 (K=104)
Profile 87 (K=106)
Profile 88 (K=159)
Profile 89 (K=246)
Profile 90 (K=109)
Profile 91 (K=133)
Profile 92 (K=115)
Profile 93 (K=323)
Profile 94 (K=251)
Profile 95 (K=211)
Profile 96 (K=151)
Profile 97 (K=139)
Profile 98 (K=467)
Profile 99 (K=152)
Profile 100 (K=126)
Profile 101 (K=144)
Profile 102 (K=187)
Profile 103 (K=142)
Profile 104 (K=100)
Profile 105 (K=223)
Profile 106 (K=196)
Profile 107 (K=231)
Profile 108 (K=286)
Profile 109 (K=153)
Profile 110 (K=129)
Profile 111 (K=136)
Profile 112 (K=105)
Profile 113 (K=104)
Profile 114 (K=115)
Profile 115 (K=100)
Profile 116 (K=145)
Profile 117 (K=152)
Profile 118 (K=113)
Profile 119 (K=137)
Profile 120 (K=129)
Profile 121 (K=101)
Profile 122 (K=109)
Profile 123 (K=184)
Profile 124 (K=216)
Profile 125 (K=111)

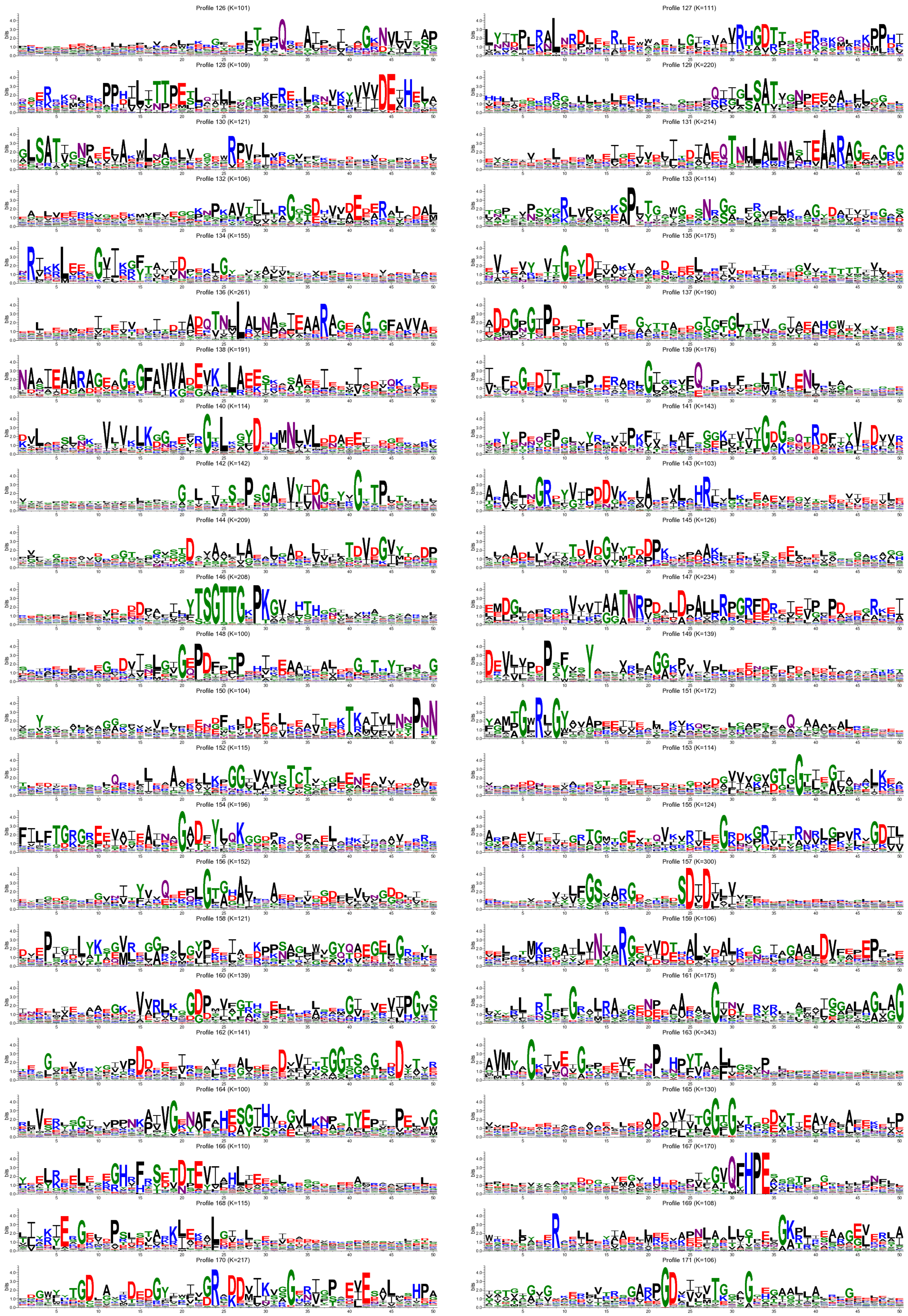
Profile 126 (K=101)
Profile 127 (K=111)
Profile 128 (K=109)
Profile 129 (K=220)
Profile 130 (K=121)
Profile 131 (K=214)
Profile 132 (K=106)
Profile 133 (K=114)
Profile 134 (K=155)
Profile 135 (K=175)
Profile 136 (K=261)
Profile 137 (K=190)
Profile 138 (K=191)
Profile 139 (K=176)
Profile 140 (K=114)
Profile 141 (K=143)
Profile 142 (K=142)
Profile 143 (K=103)
Profile 144 (K=209)
Profile 145 (K=126)
Profile 146 (K=208)
Profile 147 (K=234)
Profile 148 (K=100)
Profile 149 (K=139)
Profile 150 (K=104)
Profile 151 (K=172)
Profile 152 (K=115)
Profile 153 (K=114)
Profile 154 (K=196)
Profile 155 (K=124)
Profile 156 (K=152)
Profile 157 (K=300)
Profile 158 (K=121)
Profile 159 (K=106)
Profile 160 (K=139)
Profile 161 (K=175)
Profile 162 (K=141)
Profile 163 (K=343)
Profile 164 (K=100)
Profile 165 (K=130)
Profile 166 (K=110)
Profile 167 (K=170)
Profile 168 (K=115)
Profile 169 (K=108)
Profile 170 (K=217)
Profile 171 (K=106)

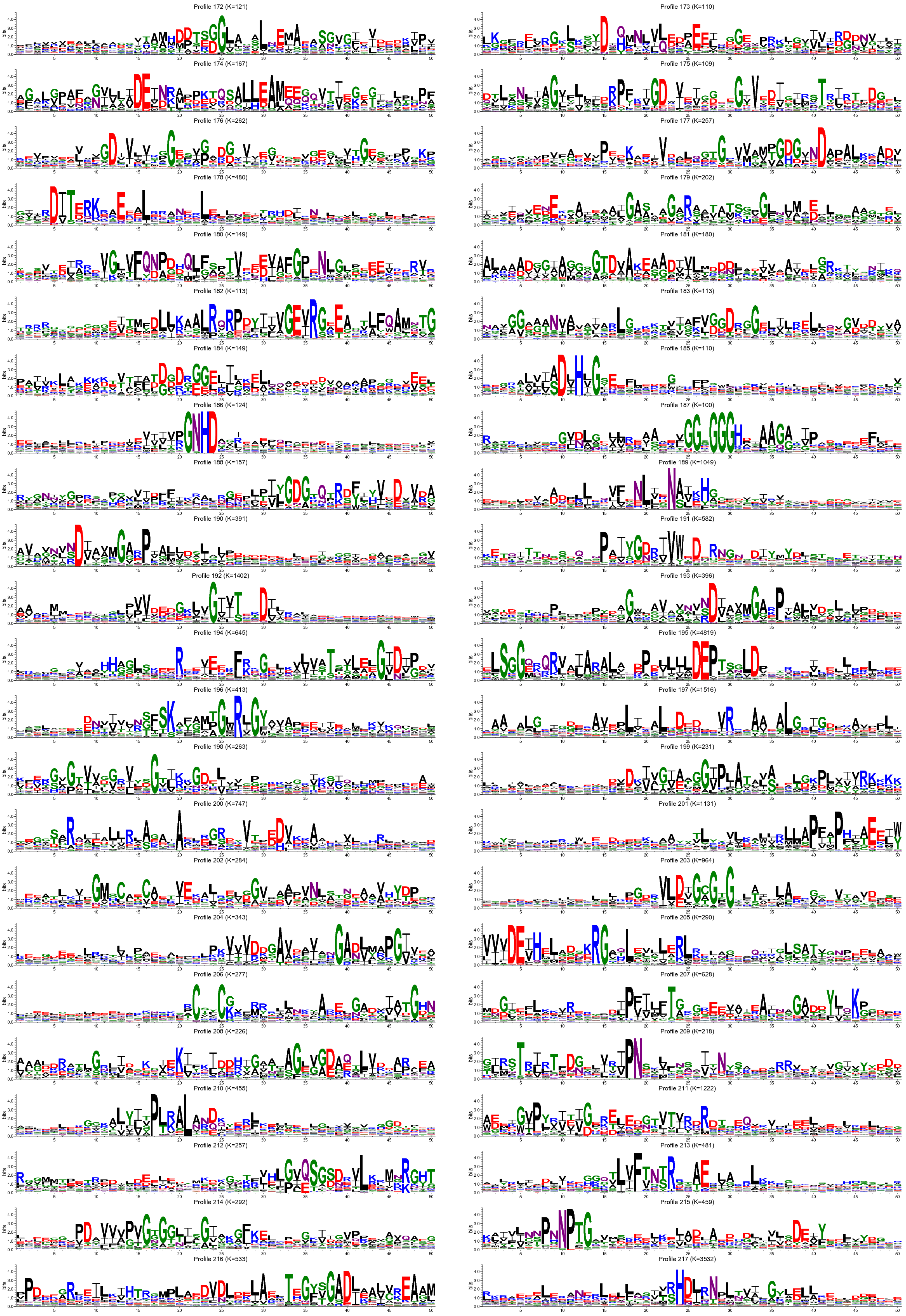
Profile 172 (K=121)
Profile 173 (K=110)
Profile 174 (K=167)
Profile 175 (K=109)
Profile 176 (K=262)
Profile 177 (K=257)
Profile 178 (K=480)
Profile 179 (K=202)
Profile 180 (K=149)
Profile 181 (K=180)
Profile 182 (K=113)
Profile 183 (K=113)
Profile 184 (K=149)
Profile 185 (K=110)
Profile 186 (K=124)
Profile 187 (K=100)
Profile 188 (K=157)
Profile 189 (K=1049)
Profile 190 (K=391)
Profile 191 (K=582)
Profile 192 (K=1402)
Profile 193 (K=396)
Profile 194 (K=645)
Profile 195 (K=4819)
Profile 196 (K=413)
Profile 197 (K=1516)
Profile 198 (K=263)
Profile 199 (K=231)
Profile 200 (K=747)
Profile 201 (K=1131)
Profile 202 (K=284)
Profile 203 (K=964)
Profile 204 (K=343)
Profile 205 (K=290)
Profile 206 (K=277)
Profile 207 (K=628)
Profile 208 (K=226)
Profile 209 (K=218)
Profile 210 (K=455)
Profile 211 (K=1222)
Profile 212 (K=257)
Profile 213 (K=481)
Profile 214 (K=292)
Profile 215 (K=459)
Profile 216 (K=533)
Profile 217 (K=3532)

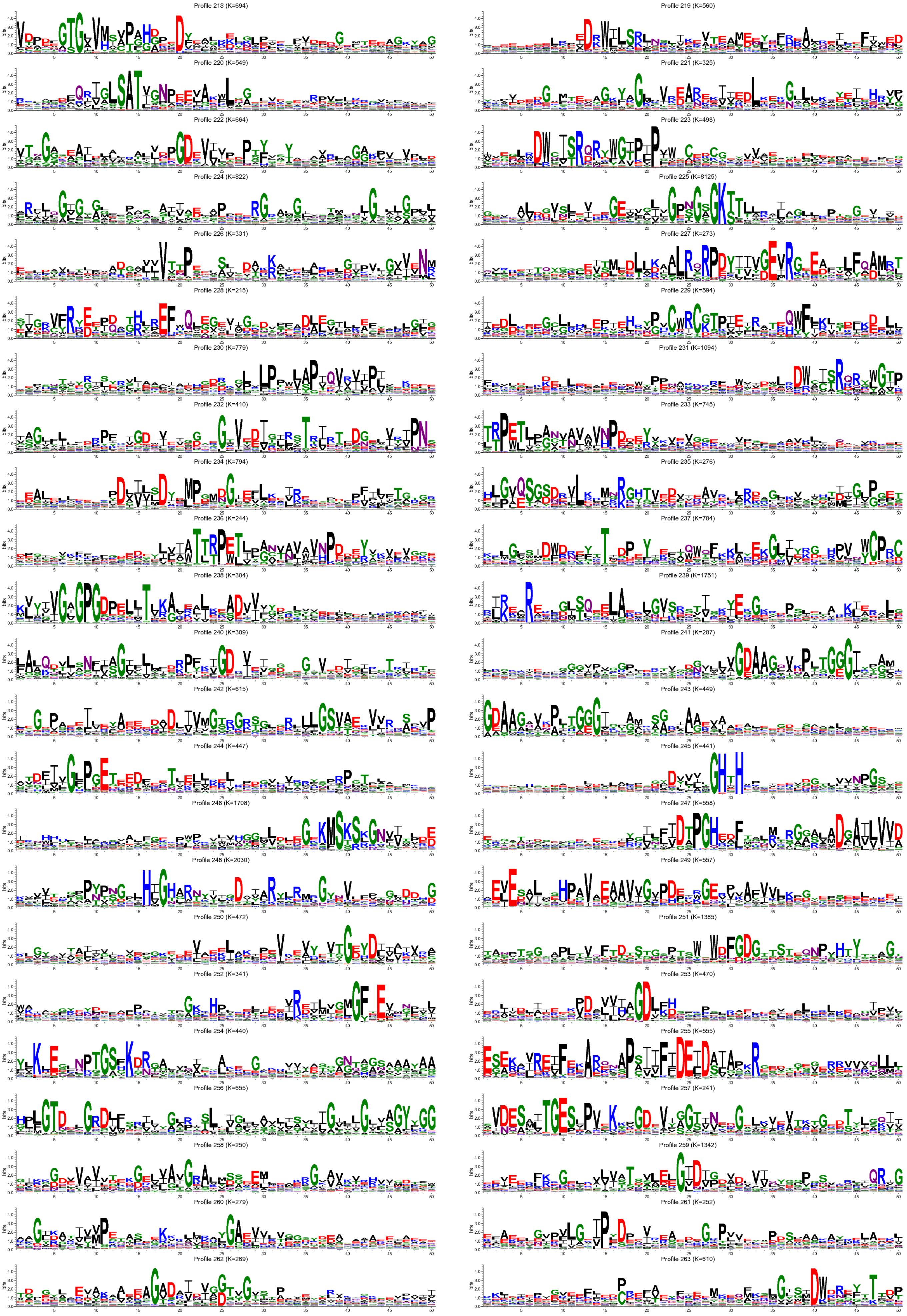
Profile 218 (K=694)
Profile 219 (K=560)
Profile 220 (K=549)
Profile 221 (K=325)
Profile 222 (K=664)
Profile 223 (K=498)
Profile 224 (K=822)
Profile 225 (K=8125)
Profile 226 (K=331)
Profile 227 (K=273)
Profile 228 (K=215)
Profile 229 (K=594)
Profile 230 (K=779)
Profile 231 (K=1094)
Profile 232 (K=410)
Profile 233 (K=745)
Profile 234 (K=794)
Profile 235 (K=276)
Profile 236 (K=244)
Profile 237 (K=784)
Profile 238 (K=304)
Profile 239 (K=1751)
Profile 240 (K=309)
Profile 241 (K=287)
Profile 242 (K=615)
Profile 243 (K=449)
Profile 244 (K=447)
Profile 245 (K=441)
Profile 246 (K=1708)
Profile 247 (K=558)
Profile 248 (K=2030)
Profile 249 (K=557)
Profile 250 (K=472)
Profile 251 (K=1385)
Profile 252 (K=341)
Profile 253 (K=470)
Profile 254 (K=440)
Profile 255 (K=555)
Profile 256 (K=655)
Profile 257 (K=241)
Profile 258 (K=250)
Profile 259 (K=1342)
Profile 260 (K=279)
Profile 261 (K=252)
Profile 262 (K=269)
Profile 263 (K=610)

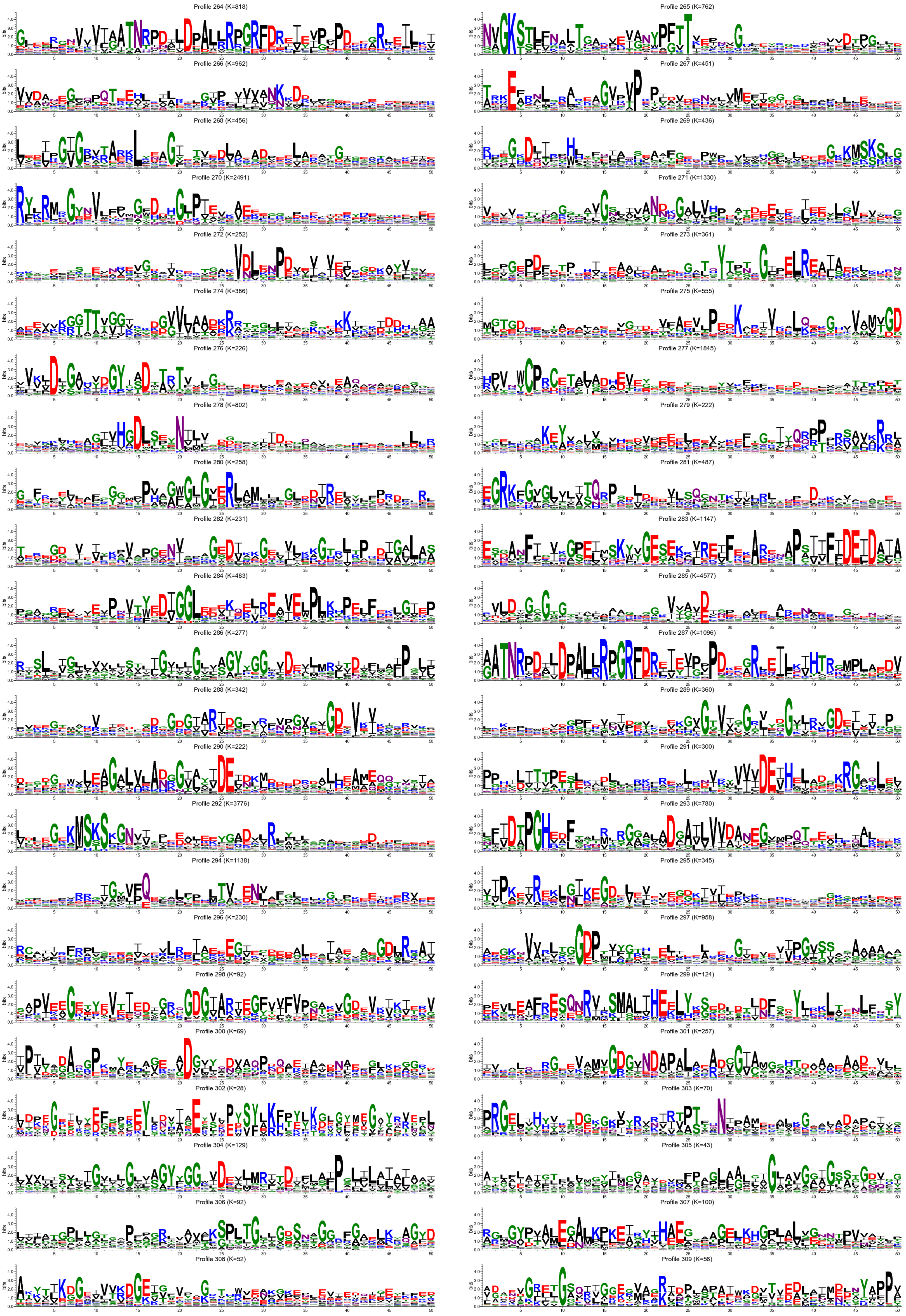

Profile 264 (K=818)
Profile 265 (K=762)
Profile 266 (K=962)
Profile 267 (K=451)
Profile 268 (K=456)
Profile 269 (K=436)
Profile 270 (K=2491)
Profile 271 (K=1330)
Profile 272 (K=252)
Profile 273 (K=361)
Profile 274 (K=386)
Profile 275 (K=555)
Profile 276 (K=226)
Profile 277 (K=1845)
Profile 278 (K=802)
Profile 279 (K=222)
Profile 280 (K=258)
Profile 281 (K=487)
Profile 282 (K=231)
Profile 283 (K=1147)
Profile 284 (K=483)
Profile 285 (K=4577)
Profile 286 (K=277)
Profile 287 (K=1096)
Profile 288 (K=342)
Profile 289 (K=360)
Profile 290 (K=222)
Profile 291 (K=300)
Profile 292 (K=3776)
Profile 293 (K=780)
Profile 294 (K=1138)
Profile 295 (K=345)
Profile 296 (K=230)
Profile 297 (K=958)
Profile 298 (K=92)
Profile 299 (K=124)
Profile 300 (K=69)
Profile 301 (K=257)
Profile 302 (K=28)
Profile 303 (K=70)
Profile 304 (K=129)
Profile 305 (K=43)
Profile 306 (K=92)
Profile 307 (K=100)
Profile 308 (K=52)
Profile 309 (K=56)

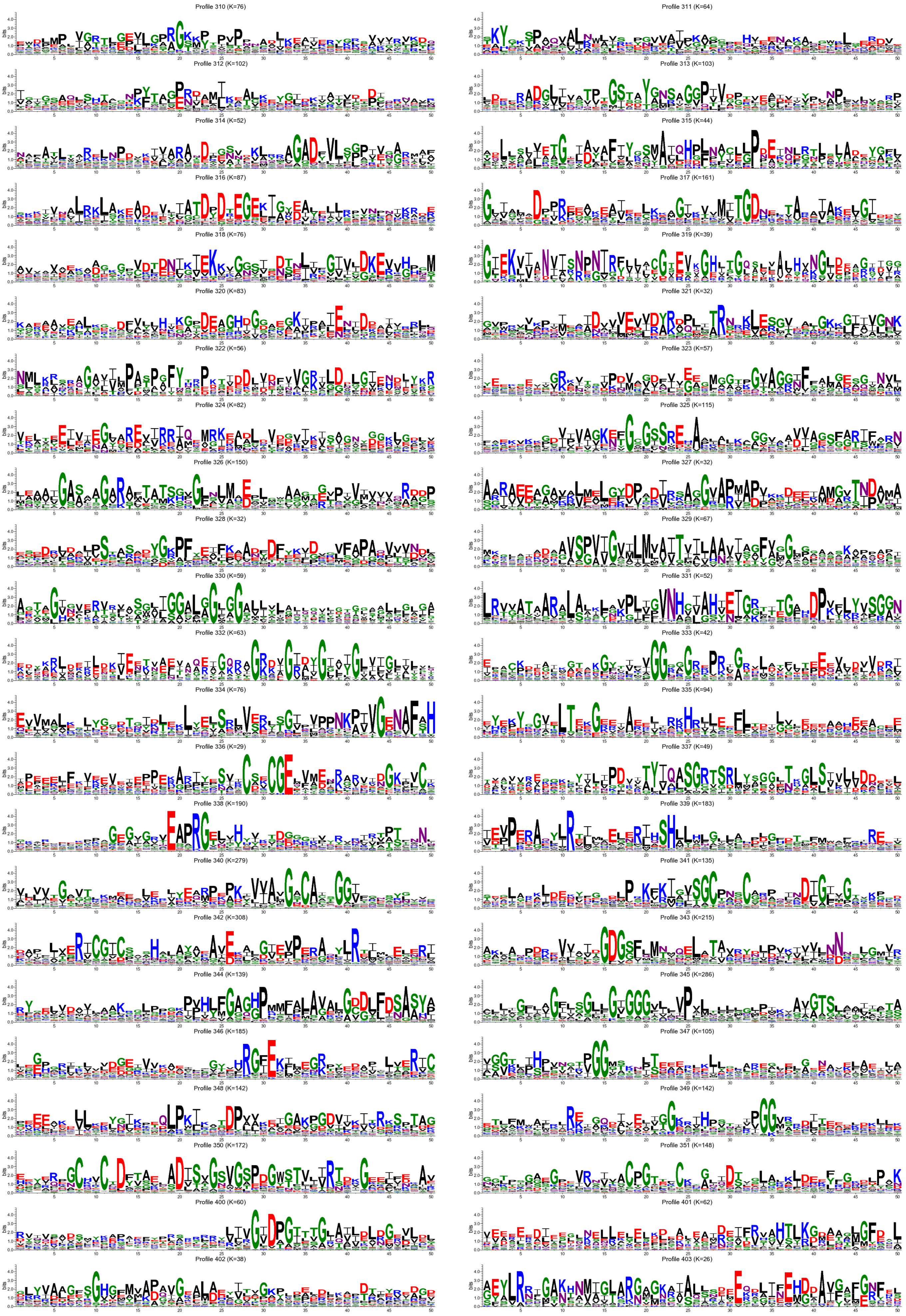
Profile 310 (K=76)
Profile 311 (K=64)
Profile 312 (K=102)
Profile 313 (K=103)
Profile 314 (K=52)
Profile 315 (K=44)
Profile 316 (K=87)
Profile 317 (K=161)
Profile 318 (K=76)
Profile 319 (K=39)
Profile 320 (K=83)
Profile 321 (K=32)
Profile 322 (K=56)
Profile 323 (K=57)
Profile 324 (K=82)
Profile 325 (K=115)
Profile 326 (K=150)
Profile 327 (K=32)
Profile 328 (K=32)
Profile 329 (K=67)
Profile 330 (K=59)
Profile 331 (K=52)
Profile 332 (K=63)
Profile 333 (K=42)
Profile 334 (K=76)
Profile 335 (K=94)
Profile 336 (K=29)
Profile 337 (K=49)
Profile 338 (K=190)
Profile 339 (K=183)
Profile 340 (K=279)
Profile 341 (K=135)
Profile 342 (K=308)
Profile 343 (K=215)
Profile 344 (K=139)
Profile 345 (K=286)
Profile 346 (K=185)
Profile 347 (K=105)
Profile 348 (K=142)
Profile 349 (K=142)
Profile 350 (K=172)
Profile 351 (K=148)
Profile 400 (K=60)
Profile 401 (K=62)
Profile 402 (K=38)
Profile 403 (K=26)

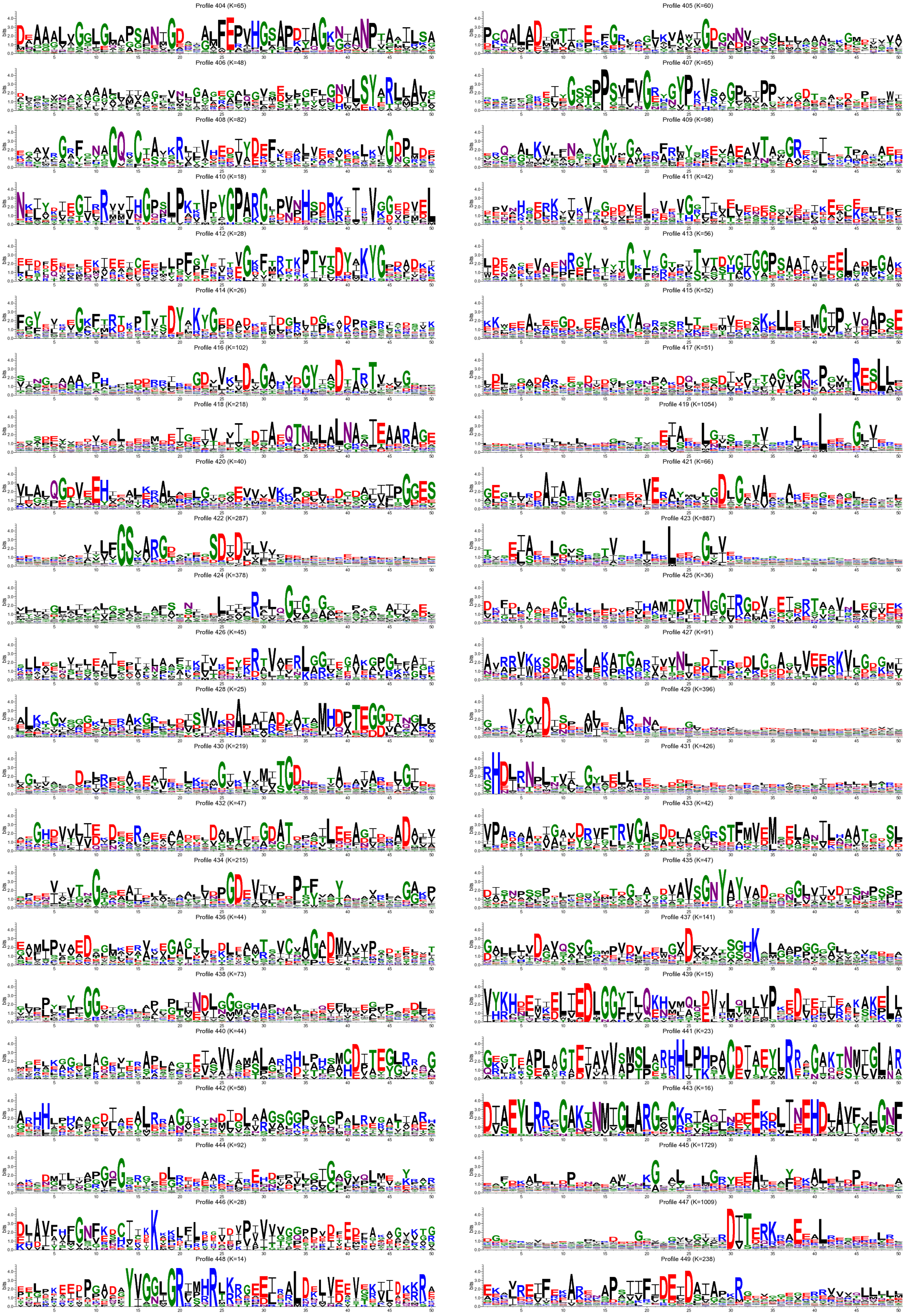
Profile 404 (K=65)
Profile 405 (K=60)
Profile 406 (K=48)
Profile 407 (K=65)
Profile 408 (K=82)
Profile 409 (K=98)
Profile 410 (K=18)
Profile 411 (K=42)
Profile 412 (K=28)
Profile 413 (K=56)
Profile 414 (K=26)
Profile 415 (K=52)
Profile 416 (K=102)
Profile 417 (K=51)
Profile 418 (K=218)
Profile 419 (K=1054)
Profile 420 (K=40)
Profile 421 (K=66)
Profile 422 (K=287)
Profile 423 (K=887)
Profile 424 (K=378)
Profile 425 (K=36)
Profile 426 (K=45)
Profile 427 (K=91)
Profile 428 (K=25)
Profile 429 (K=396)
Profile 430 (K=219)
Profile 431 (K=426)
Profile 432 (K=47)
Profile 433 (K=42)
Profile 434 (K=215)
Profile 435 (K=47)
Profile 436 (K=44)
Profile 437 (K=141)
Profile 438 (K=73)
Profile 439 (K=15)
Profile 440 (K=44)
Profile 441 (K=23)
Profile 442 (K=58)
Profile 443 (K=16)
Profile 444 (K=92)
Profile 445 (K=1729)
Profile 446 (K=28)
Profile 447 (K=1009)
Profile 448 (K=14)
Profile 449 (K=238)

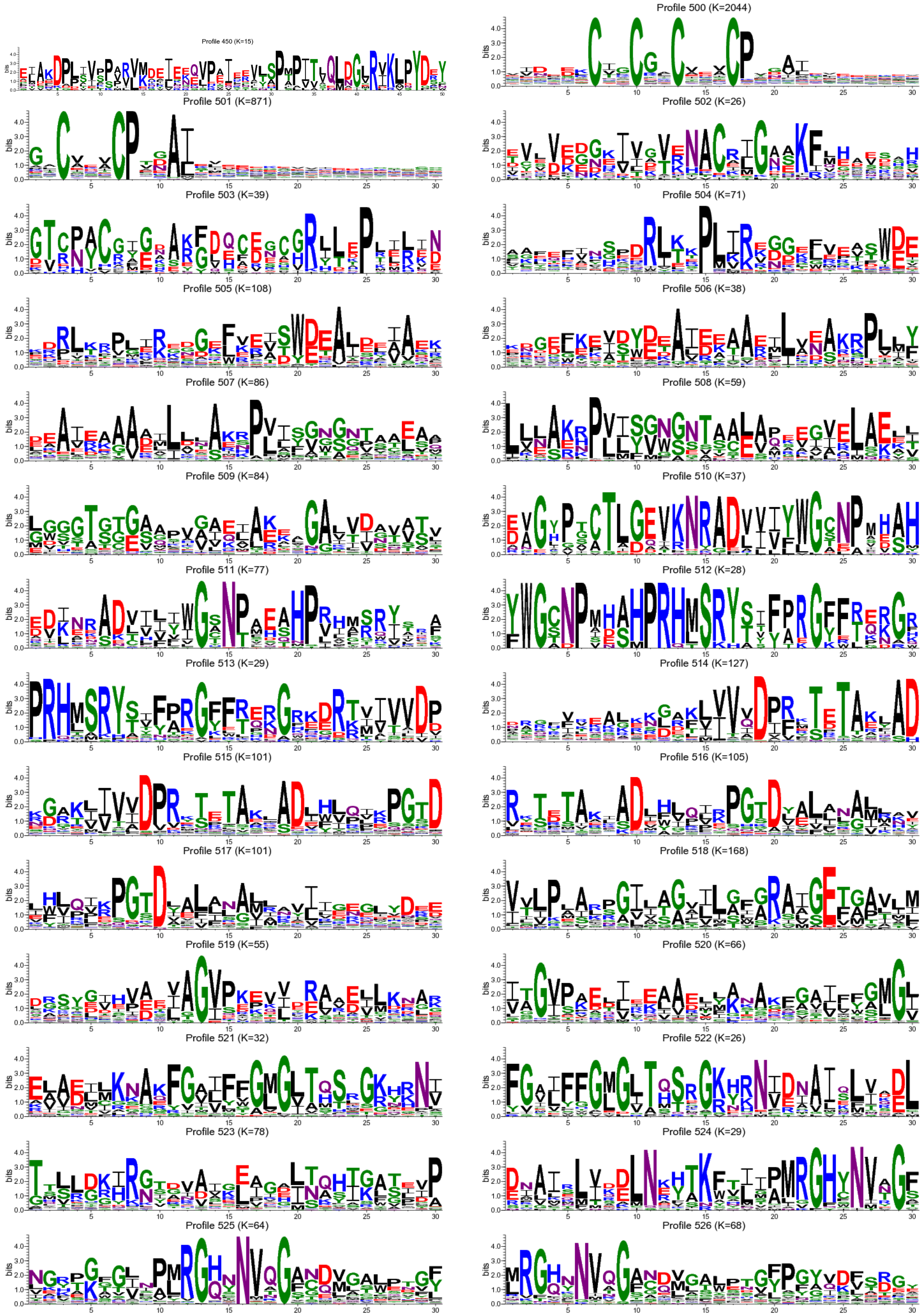
Profile 450 (K=15)
Profile 500 (K=2044)
Profile 501 (K=871)
Profile 502 (K=26)
Profile 503 (K=39)
Profile 504 (K=71)
Profile 505 (K=108)
Profile 506 (K=38)
Profile 507 (K=86)
Profile 508 (K=59)
Profile 509 (K=84)
Profile 510 (K=37)
Profile 511 (K=77)
Profile 512 (K=28)
Profile 513 (K=29)
Profile 514 (K=127)
Profile 515 (K=101)
Profile 516 (K=105)
Profile 517 (K=101)
Profile 518 (K=168)
Profile 519 (K=55)
Profile 520 (K=66)
Profile 521 (K=32)
Profile 522 (K=26)
Profile 523 (K=78)
Profile 524 (K=29)
Profile 525 (K=64)
Profile 526 (K=68)

Profile 527 (K=38)

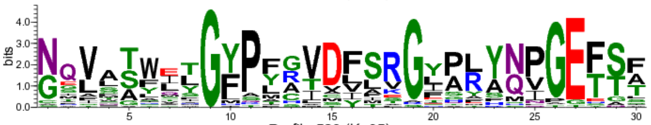

Profile 528 (K=53)

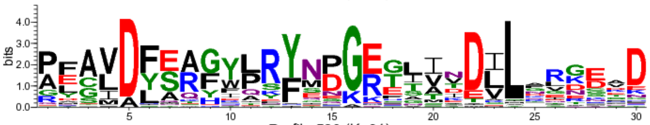

Profile 529 (K=35)

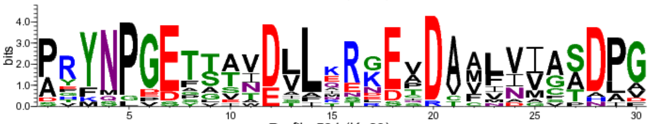

Profile 530 (K=31)

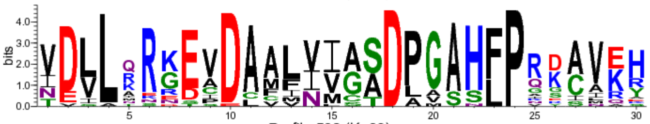

Profile 531 (K=29)

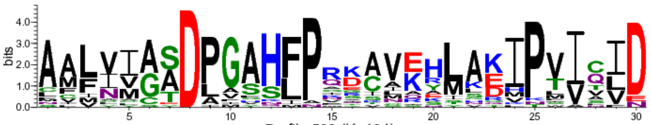

Profile 532 (K=28)

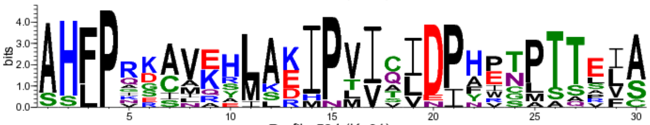

Profile 533 (K=184)

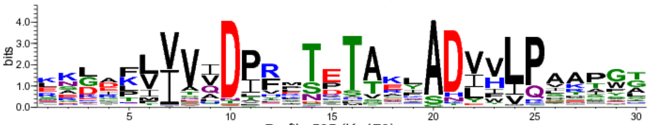

Profile 534 (K=31)

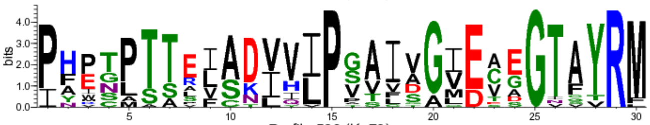

Profile 535 (K=179)

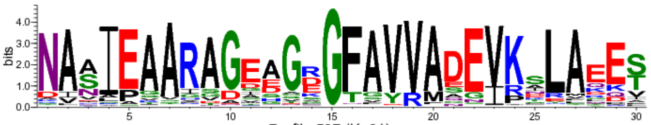

Profile 536 (K=79)

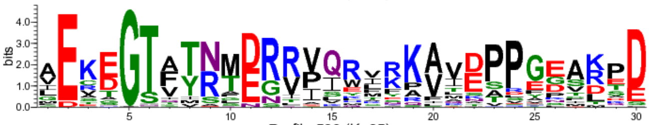

Profile 537 (K=81)

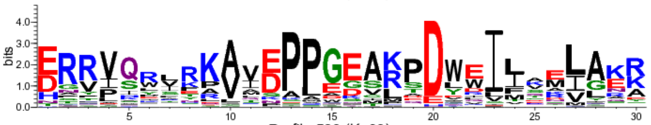

Profile 538 (K=85)

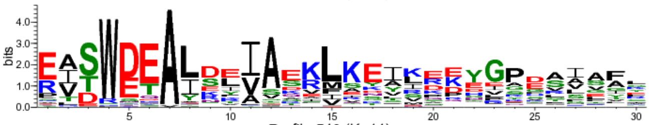

Profile 539 (K=39)

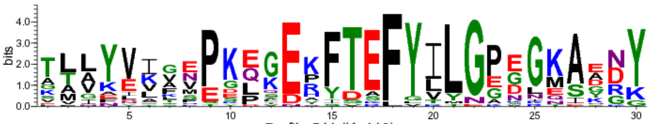

Profile 540 (K=14)

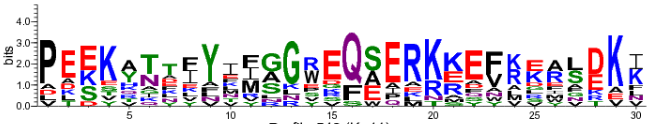

Profile 541 (K=116)

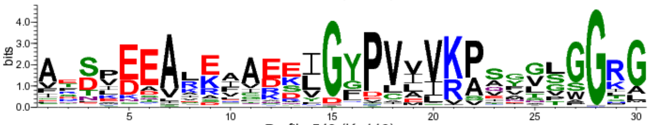

Profile 542 (K=11)

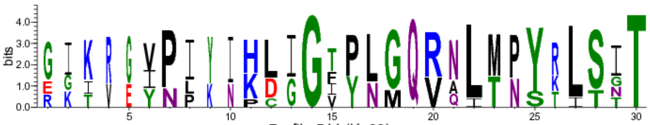

Profile 543 (K=118)

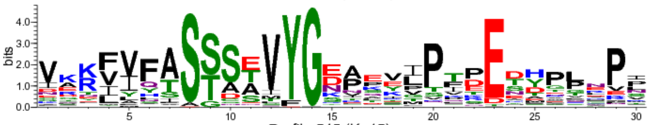

Profile 544 (K=33)

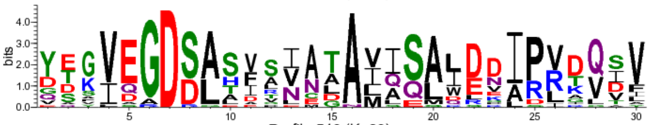

Profile 545 (K=15)

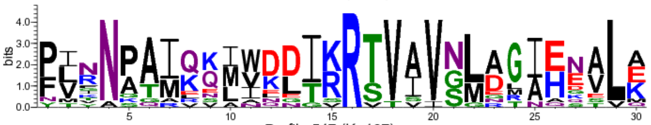

Profile 546 (K=26)

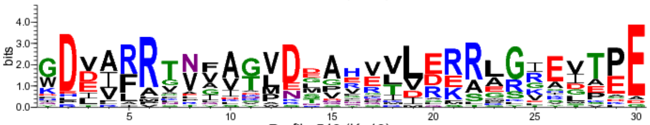

Profile 547 (K=167)

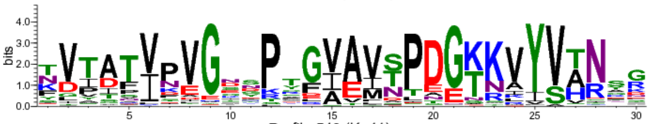

Profile 548 (K=16)

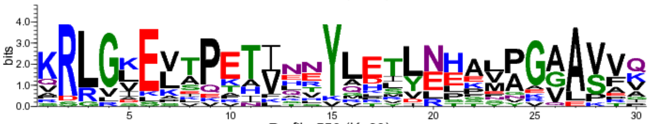

Profile 549 (K=41)

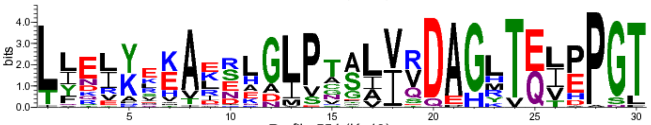

Profile 550 (K=30)

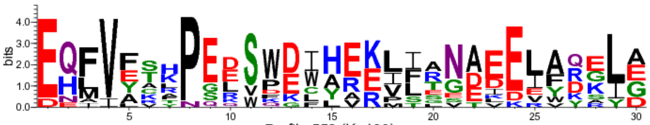

Profile 551 (K=10)

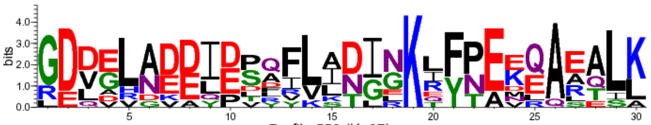

Profile 552 (K=180)

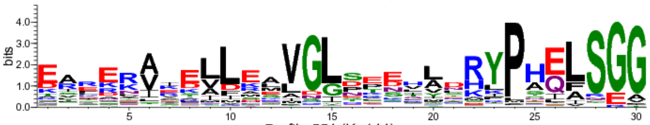

Profile 553 (K=27)

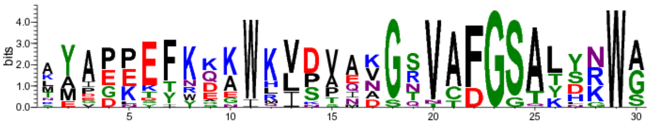

Profile 554 (K=144)

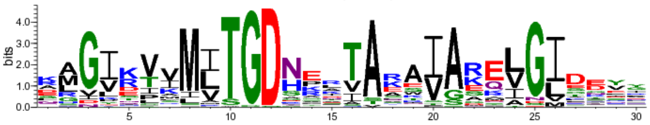

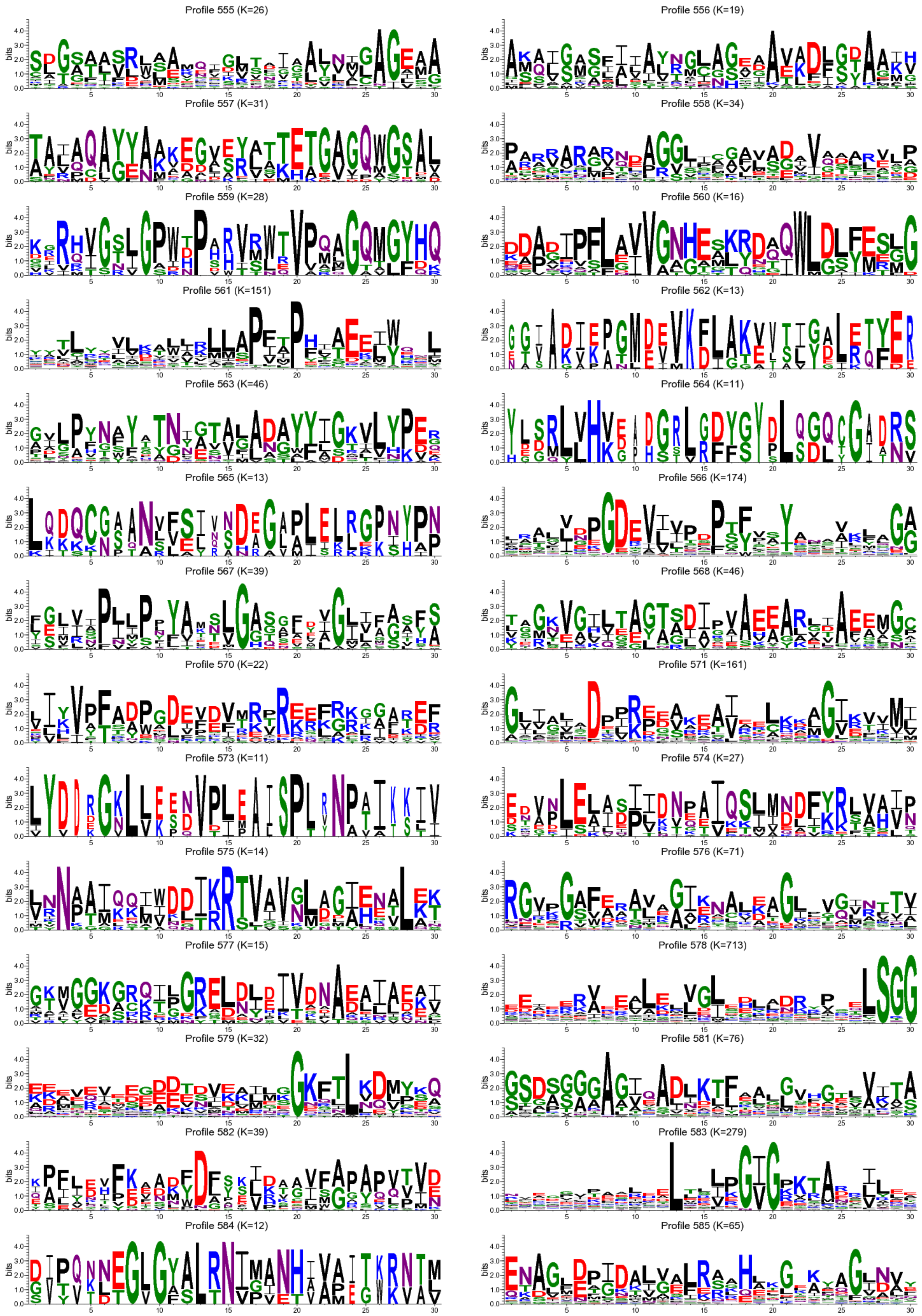
Profile 555 (K=26)
Profile 556 (K=19)
Profile 557 (K=31)
Profile 558 (K=34)
Profile 559 (K=28)
Profile 560 (K=16)
Profile 561 (K=151)
Profile 562 (K=13)
Profile 563 (K=46)
Profile 564 (K=11)
Profile 565 (K=13)
Profile 566 (K=174)
Profile 567 (K=39)
Profile 568 (K=46)
Profile 570 (K=22)
Profile 571 (K=161)
Profile 573 (K=11)
Profile 574 (K=27)
Profile 575 (K=14)
Profile 576 (K=71)
Profile 577 (K=15)
Profile 578 (K=713)
Profile 579 (K=32)
Profile 581 (K=76)
Profile 582 (K=39)
Profile 583 (K=279)
Profile 584 (K=12)
Profile 585 (K=65)
bits

Profile 586 (K=17)

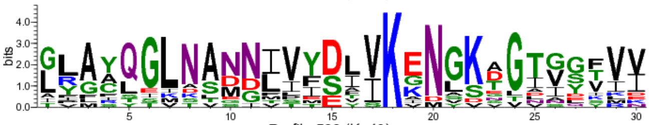

Profile 587 (K=64)

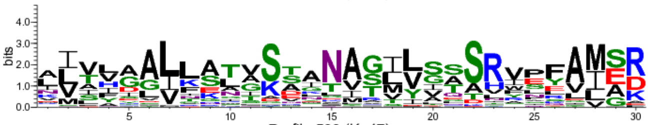

Profile 588 (K=40)

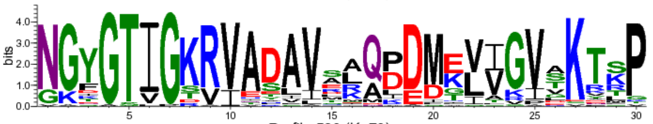

Profile 589 (K=47)

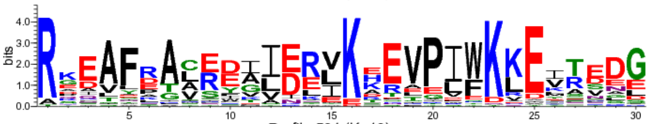

Profile 590 (K=70)

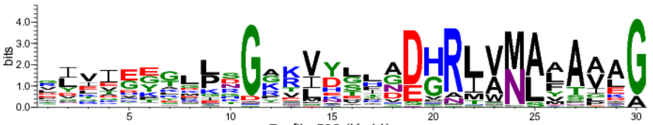

Profile 591 (K=16)

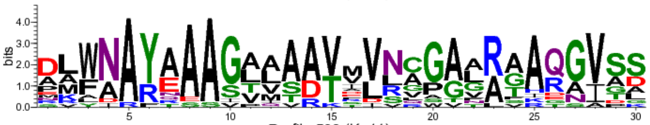

Profile 592 (K=14)

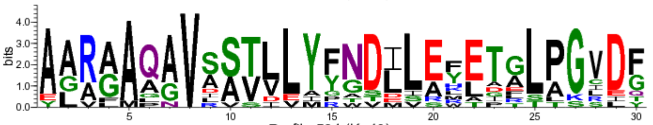

Profile 593 (K=11)

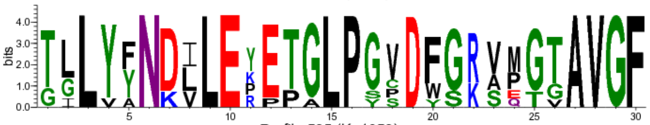

Profile 594 (K=40)

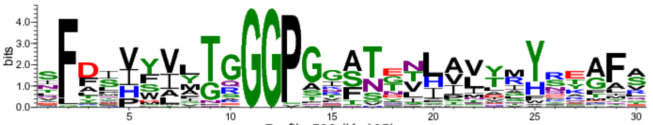

Profile 595 (K=1053)

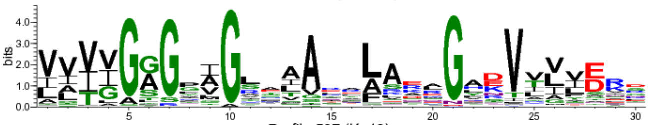

Profile 596 (K=165)

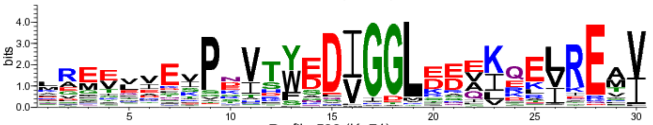

Profile 597 (K=18)

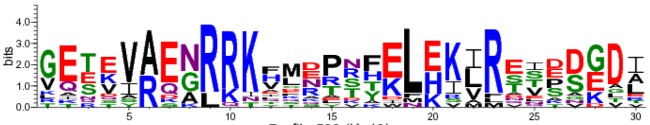

Profile 598 (K=74)

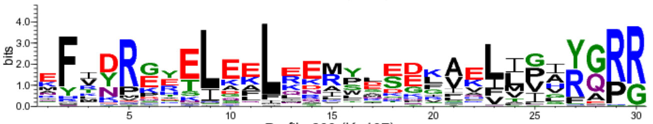

Profile 599 (K=10)

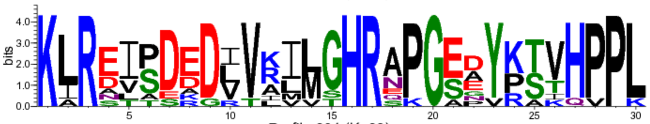

Profile 600 (K=107)

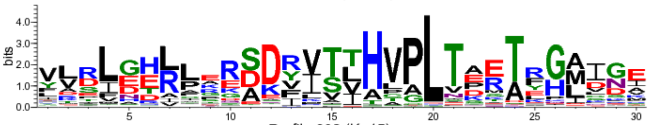

Profile 601 (K=33)

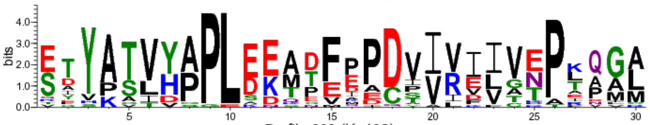

Profile 602 (K=15)

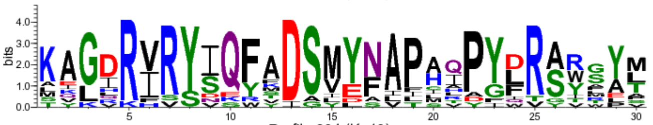

Profile 603 (K=192)

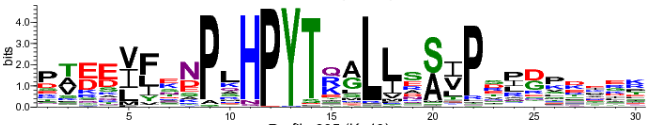

Profile 604 (K=10)

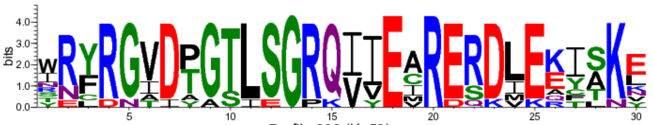

Profile 605 (K=10)

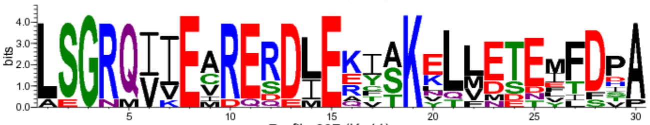

Profile 606 (K=53)

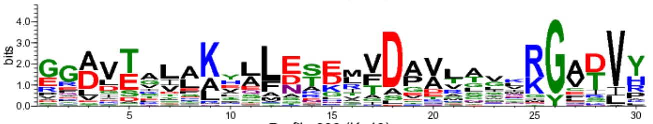

Profile 607 (K=11)

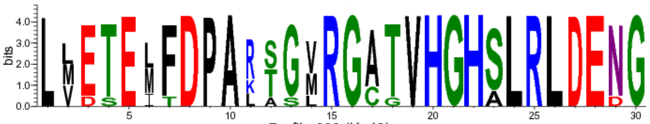

Profile 608 (K=10)

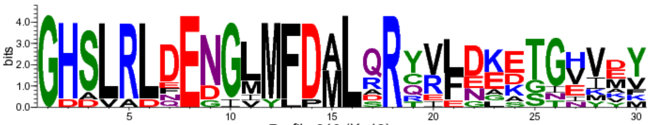

Profile 609 (K=48)

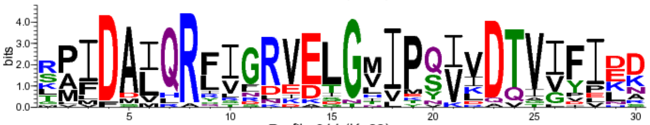

Profile 610 (K=18)

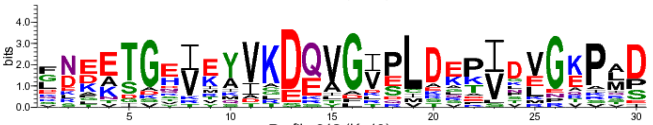

Profile 611 (K=22)

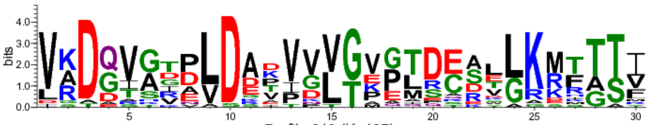

Profile 612 (K=10)

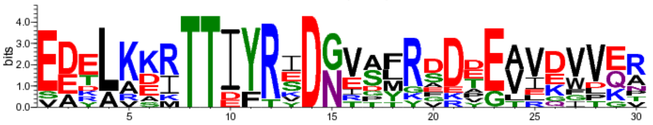

Profile 613 (K=127)

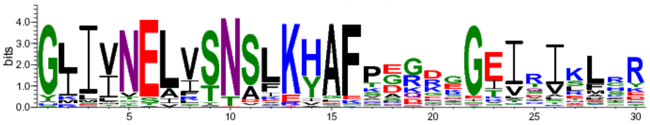

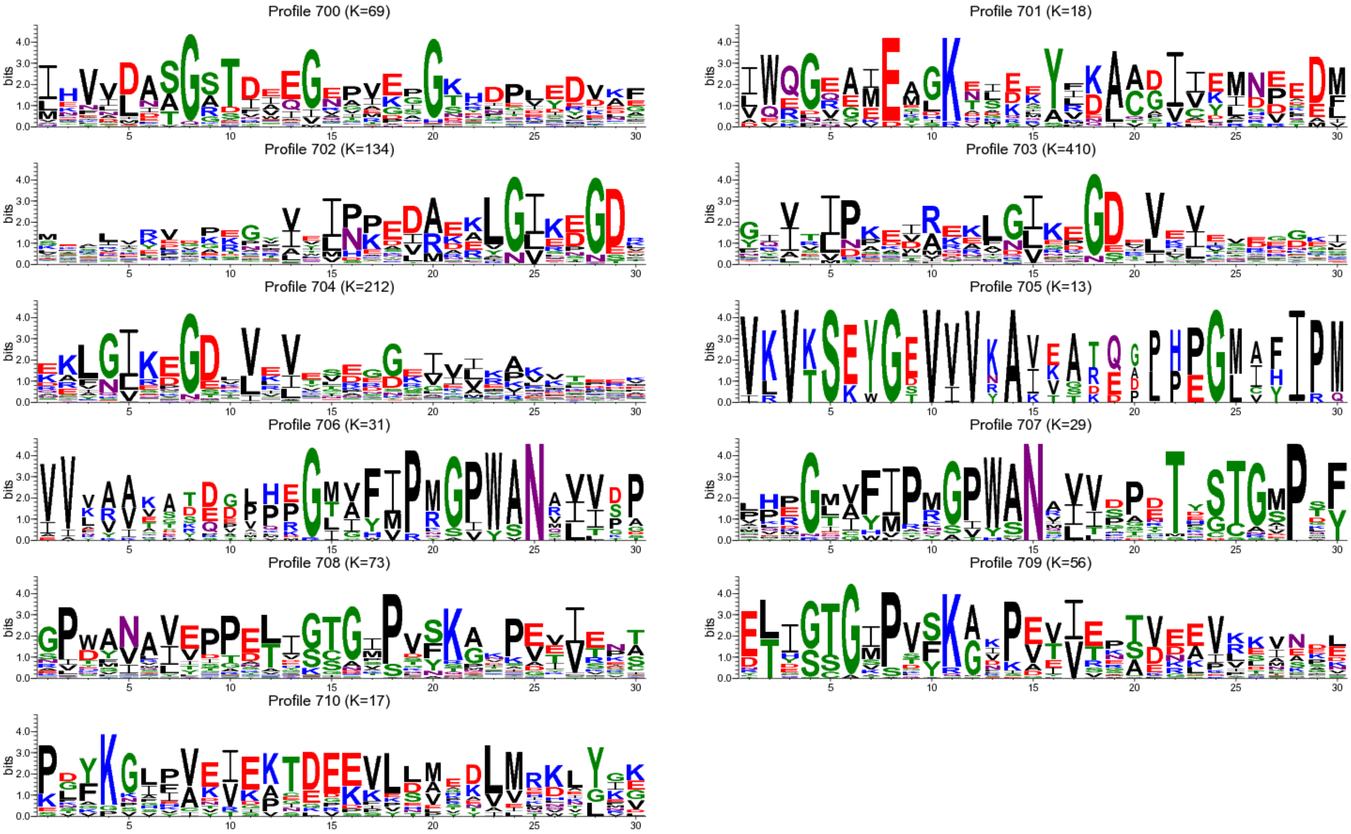
Profile 700 (K=69)
Profile 701 (K=18)
Profile 702 (K=134)
Profile 703 (K=410)
Profile 704 (K=212)
Profile 705 (K=13)
Profile 706 (K=31)
Profile 707 (K=29)
Profile 708 (K=73)
Profile 709 (K=56)
Profile 710 (K=17)
