# Supplementary material for: Exploring the evolution of protein function in Archaea
Source: BMC Evol Biol. 2012 May 30;12:75. doi: 10.1186/1471-2148-12-75 (PMC3458885; doi:10.1186/1471-2148-12-75)
Supplement: Additional file 3 — Contains description of enzymes involved in methanogenesis, the list of the corresponding arCOGs (Additional file3: Table S1), and the graphs of connections between methanogenic arCOGs (Additional file3: Figure S1) and the domains comprising them (Additional file3: Figure S2) [[29],[35],[58-61]]. [file 1471-2148-12-75-S3.pdf]

Exploring the evolution of protein function in Archaea

Alexander Goncearenco and Igor N. Berezovsky

Additional File 2

Description of steps of interest in the methanogenesis pathway

Step 1

In the first step of the methanogenic pathway the carbon is being transferred to methanofuran (MF). There are two isoforms of enzymes catalyzing the produciton of methyl-MF: a molybdenum- (Fmd) and a tungsteen-dependent formyl-MF dehydrogenase (Fwd), utilizing molybdopterin and tungsteen-pterin cofactors respectively. Usually, both the Fmd and Fwd operons are present in the genome, and the activation of one or another depends on the metal availability in the environment and on the other environmental factors. The details of the genomic organization of Fmd/Fwd enzymes is different between methanogens [1]. The subunit E of the Fmd enzyme in *M. thermautotrophicus* contains the EFL with the typical signature of DNA binding zinc fingers, which indicates that FmdE potentially works as a transcription factor activating the metal-dependent genes[2]. In *M. jannaschii* Fwd subunit A is a metallo-dependent hydrolase and presumably has a β/α-barrel fold. Fwd subunit F is a polyferredoxin, which is characterized by the presence of eight [4Fe-4S]-cluster binding sites creating an electron transfer cascade. Fwd subunits B and D are involved in binding molybdopterin cofactor. Despite the name of the cofactor, it contains the tungsten atom instead of the molybdenum. FwdB is the central catalytic subunit of the enzyme universally present in all methanogenes. It belongs to a molybdopterin binding superfamily (MopB in CDD), which is characterized by the presence of the molybdopterin cofactor (e.g. molybdopterin

guanidine dinucleotide, MGD) in the catalysis. This superfamily also includes other biochemical functions, such as formate dehydrogenase (FdhH, FdhN) and dimethylsulfoxide reductase (DMSO). It has been shown[3] that the structure and mechanism of the biochemical reaction in molybdopterin-utilizing enzymes, especially in di(MGD) utilizing enzymes, are conserved. Therefore, we use an available structure of the Formate dehydrogenase H from *E. coli* (FdhH) as the model for FmdB.

Step 7

Methyl-coenzyme M reductase (Mcr) catalyzes the last, 7th step of methanogenesis – reduction of the methyl-CoM with coenzyme B (CoB) yielding methane. The cofactor F_{430} is a key element defining the Mcr function, because its electrochemical properties are essential for driving the reaction. An evolutionary history of the cofactor F_{430} helps to understand the very origins of the Mcr. It has been suggested that the modified tetrapyrroles (including F_{430}) can have an early, even prebiotic, evolutionary origin[4]. In general, tetrapyrroles, such as hemes, chlorophil, and cobalamin are wide-spread and utilized in different biochemical processes. The F_{430}, however, has a very specific role, and it is the only one that contains nickel. It is also the most reduced one, suggesting that F_{430} requires strictly anaerobic conditions. This cofactor has only been found in connection to Mcr enzyme in methanogenic archaea and in some anaerobic bacteria. The Mcr was presumably formed first in methanogenic archaea and then via lateral gene transfer was acquired by methanotrophic bacteria, where it catalyzes the reverse reaction[5]. The crystal structure of Mcr is available – it is a hexamer consisting of two alpha, two beta, and two gamma subunits coordinating two F_{430} cofactor molecules[6]. The F_{430} is buried deep in the enzyme, and two substrates (CoM-SH and CoB-SH) enter the channel leading to the cofactor.

Step connecting steps 1 and 7: heterosulfide reductase (Hdr)

Enzyme heterosulfide reductase (Hdr) is an example of the multiple utilization of one and the same elementary function. Hdr provides reduced ferredoxin for the Fmd/Fwd enzyme (the first step in methanogenic pathway). At the same time, Hdr catalyzes reduction of the heterosulfide CoM-S-S-CoB to CoM-SH and HS-CoB used by the Mcr enzyme (the last step of methanogenesis).

References

1. Ferry JG: **Enzymology of one-carbon metabolism in methanogenic pathways.** *FEMS Microbiol Rev* 1999, **23:**13-38.
2. Axelrod HL, Das D, Abdubek P, Astakhova T, Bakolitsa C, Carlton D, Chen C, Chiu HJ, Clayton T, Deller MC, et al: **Structures of three members of Pfam PF02663 (FmdE) implicated in microbial methanogenesis reveal a conserved alpha+beta core domain and an auxiliary C-terminal treble-clef zinc finger.** *Acta Crystallogr Sect F Struct Biol Cryst Commun* 2010, **66:**1335-1346.
3. Schindelin H, Kisker C, Hilton J, Rajagopalan KV, Rees DC: **Crystal structure of DMSO reductase: redox-linked changes in molybdopterin coordination.** *Science* 1996, **272:**1615-1621.
4. Holliday GL, Thornton JM, Marquet A, Smith AG, Rebeille F, Mendel R, Schubert HL, Lawrence AD, Warren MJ: **Evolution of enzymes and pathways for the biosynthesis of cofactors.** *Nat Prod Rep* 2007, **24:**972-987.
5. Scheller S, Goenrich M, Boecher R, Thauer RK, Jaun B: **The key nickel enzyme of methanogenesis catalyses the anaerobic oxidation of methane.** *Nature* 2010, **465:**606-608.
6. Ermler U, Grabarse W, Shima S, Goubeaud M, Thauer RK: **Crystal structure of methyl-coenzyme M reductase: the key enzyme of biological methane formation.** *Science* 1997, **278:**1457-1462.

Table ST1. Central enzymes in the CO_2 methanogenesis pathway and their subunits; the corresponding COGs and arCOGs

Step	EC	Name	Subunit	COG	COG name	arCOG	arCOG name
1	1.2.99.5	Fmd/ Fwd	*				
			fmdA	COG1229	Formylmethanofuran dehydrogenase subunit A	arCOG04461	Formylmethanofuran dehydrogenase subunit A
			fmdB	COG1029	Formylmethanofuran dehydrogenase subunit B		
						arCOG01498	Formylmethanofuran dehydrogenase subunit B
						arCOG01499	Formylmethanofuran dehydrogenase subunit B
			fmdC	COG2218	Formylmethanofuran dehydrogenase subunit C		
						arCOG00097	Formylmethanofuran dehydrogenase subunit C
						arCOG00098	Formylmethanofuran dehydrogenase subunit C
			fmdD	COG1153	Formylmethanofuran dehydrogenase subunit D	arCOG02674	Formylmethanofuran dehydrogenase subunit D
			fmdH	COG1146	Ferredoxin		
						arCOG00959	Ferredoxin
						arCOG00960	Ferredoxin
						arCOG02461	Ferredoxin
						arCOG02587	Ferredoxin
						arCOG02618	Ferredoxin
						arCOG04548	Ferredoxin
						arCOG04549	Uncharacterized conserved protein
						arCOG04550	Uncharacterized conserved protein
						arCOG04551	Uncharacterized conserved protein
						arCOG04891	Ferredoxin fused to HTH-domain transciptional regulator
						arCOG04892	Uncharacterized conserved protein
			fmdF	COG1145	Ferredoxin		
						arCOG00291	Ferredoxin
						arCOG00292	Ferredoxin domain containing protein
						arCOG00296	Ferredoxin
						arCOG00300	Ferredoxin
						arCOG00958	Ferredoxin
						arCOG00961	Ferredoxin
						arCOG01610	Ferredoxin
						arCOG01611	Ferredoxin
						arCOG02060	Ferredoxin
						arCOG02179	Polyferredoxin
						arCOG02180	Polyferredoxin
						arCOG02181	Polyferredoxin
						arCOG02182	Polyferredoxin
						arCOG02183	Polyferredoxin

						arCOG02184	Polyferredoxin
						arCOG02185	Polyferredoxin
						arCOG02187	Ferredoxin domain containing protein
						arCOG02189	HTH containing ranscriptional regulator fused to ferredoxin domain
						arCOG02237	Polyferredoxin
						arCOG02445	Ferredoxin
						arCOG02449	Flavodoxin fused to ferredoxin domain
						arCOG02451	Ferredoxin fused to a conserved domain of DUF362 family
						arCOG02452	Uncharacterized conserved protein
						arCOG02460	Ferredoxin
						arCOG02588	Ferredoxin
						arCOG02619	Ferredoxin
						arCOG04074	Ferredoxin
						arCOG04476	Ferredoxin
						arCOG05128	Ferredoxin
						arCOG05466	Ferredoxin domain containing protein
						arCOG05467	Uncharacterized conserved protein
						arCOG06908	Ferredoxin
						arCOG07877	UbiA prenyltransferase family enzyme
			fmdE	COG2191	Formylmethanofuran dehydrogenase subunit E		
						arCOG00762	Formylmethanofuran dehydrogenase subunit E
						arCOG00763	Formylmethanofuran dehydrogenase subunit E
						arCOG00764	Formylmethanofuran dehydrogenase subunit E
						arCOG07877	UbiA prenyltransferase family enzyme
			fwdG	COG1145	Ferredoxin	(see above)	
2	2.3.1.101	Ftr	-	COG2037	Formylmethanofuran:tetrahydromethanopterin formyltransferase	arCOG02695	Formylmethanofuran:tetrahydromethanopterin formyltransferase
3	3.5.4.27	Mch	-	COG3252	Methenyltetrahydromethanopterin cyclohydrolase	arCOG02675	Methenyltetrahydromethanopterin cyclohydrolase
4	1.5.99.9	Mtd	-	COG1927	Coenzyme F420-dependent N(5),N(10)-methenyltetrahydromethanopterin dehydrogenase	arCOG04382	Coenzyme F420-dependent N(5),N(10)-methenyltetrahydromethanopterin dehydrogenase
	1.5.1._						

	1.12.98.2	Hmd	-	COG4074	H2-forming N5,N10-methylenetetrahydromethanopterin dehydrogenase		
						arCOG03196	H2-forming N5,N10-methylenetetrahydromethanopterin dehydrogenase
						arCOG03197	Uncharacterized conserved protein
5	1.5.99.11	Mer	-	COG2141	Coenzyme F420-dependent N5,N10-methylene tetrahydromethanopterin reductase and related flavin-dependent oxidoreductases	arCOG02410	Coenzyme F420-dependent N5,N10-methylene tetrahydromethanopterin reductase or related flavin-dependent oxidoreductase
6	2.1.1.86	Mtr	*				
			mtrA	COG4063	Tetrahydromethanopterin S-methyltransferase, subunit A		
						arCOG03220	Tetrahydromethanopterin S-methyltransferase, subunit A
						arCOG03221	Tetrahydromethanopterin S-methyltransferase, subunit A
			mtrB	COG4062	Tetrahydromethanopterin S-methyltransferase, subunit B	arCOG04867	Tetrahydromethanopterin S-methyltransferase, subunit B
			mtrC	COG4061	Tetrahydromethanopterin S-methyltransferase, subunit C	arCOG04868	Tetrahydromethanopterin S-methyltransferase, subunit C
			mtrD	COG4060	Tetrahydromethanopterin S-methyltransferase, subunit D	arCOG04869	Tetrahydromethanopterin S-methyltransferase, subunit D
			mtrE	COG4059	Tetrahydromethanopterin S-methyltransferase, subunit E	arCOG04870	Tetrahydromethanopterin S-methyltransferase, subunit E
			mtrF	COG4218	Tetrahydromethanopterin S-methyltransferase, subunit F		
						arCOG03381	Tetrahydromethanopterin S-methyltransferase, subunit F
						arCOG07877	UbiA prenyltransferase family enzyme
			mtrG	COG4064	Tetrahydromethanopterin S-methyltransferase, subunit G	arCOG03380	Tetrahydromethanopterin S-methyltransferase, subunit G
			mtrH	COG1962	Tetrahydromethanopterin S-methyltransferase, subunit H	arCOG04336	Tetrahydromethanopterin S-methyltransferase, subunit H

7	2.8.4.1	Mcr	*				
			mcrA	-			
			component A2	COG1123	ATPase components of various ABC-type transport systems, contain duplicated ATPase	arCOG00185	ATPase component of various ABC-type transport system, contain duplicated ATPase
			mcrB	-			
			mcrG	-		Phosphoribosyl formylglycinamidine (FGAM) synthase, synthetase domain	
			mcrC	-			
			mcrD	-			
	1.8.98.1	Hdr	*				
			hdrA	COG1148	Heterodisulfide reductase, subunit A and related polyferredoxins		
						arCOG02234	Heterodisulfide reductase, subunit A, polyferredoxin
						arCOG02235	Heterodisulfide reductase, subunit A or related polyferredoxin
						arCOG02236	Heterodisulfide reductase, subunit A or related polyferredoxin
						arCOG02476	Heterodisulfide reductase, subunit A; ferredoxin domain
						arCOG04117	Ferredoxin
						arCOG04118	Mn2+/Fe2+ transporter, NRAMP family
			hdrB	COG2048	Heterodisulfide reductase, subunit B		
						arCOG00338	Heterodisulfide reductase, subunit B
						arCOG00342	Heterodisulfide reductase, subunit B
						arCOG07877	UbiA prenyltransferase family enzyme
			hdrC	COG1150	Heterodisulfide reductase, subunit C		
						arCOG00964	Heterodisulfide reductase, subunit C
						arCOG00965	Heterodisulfide reductase, subunit C
						arCOG00966	Heterodisulfide reductase, subunit C
			hdrD	COG1150		(see above)	
			hdrE	COG1150		(see above)	

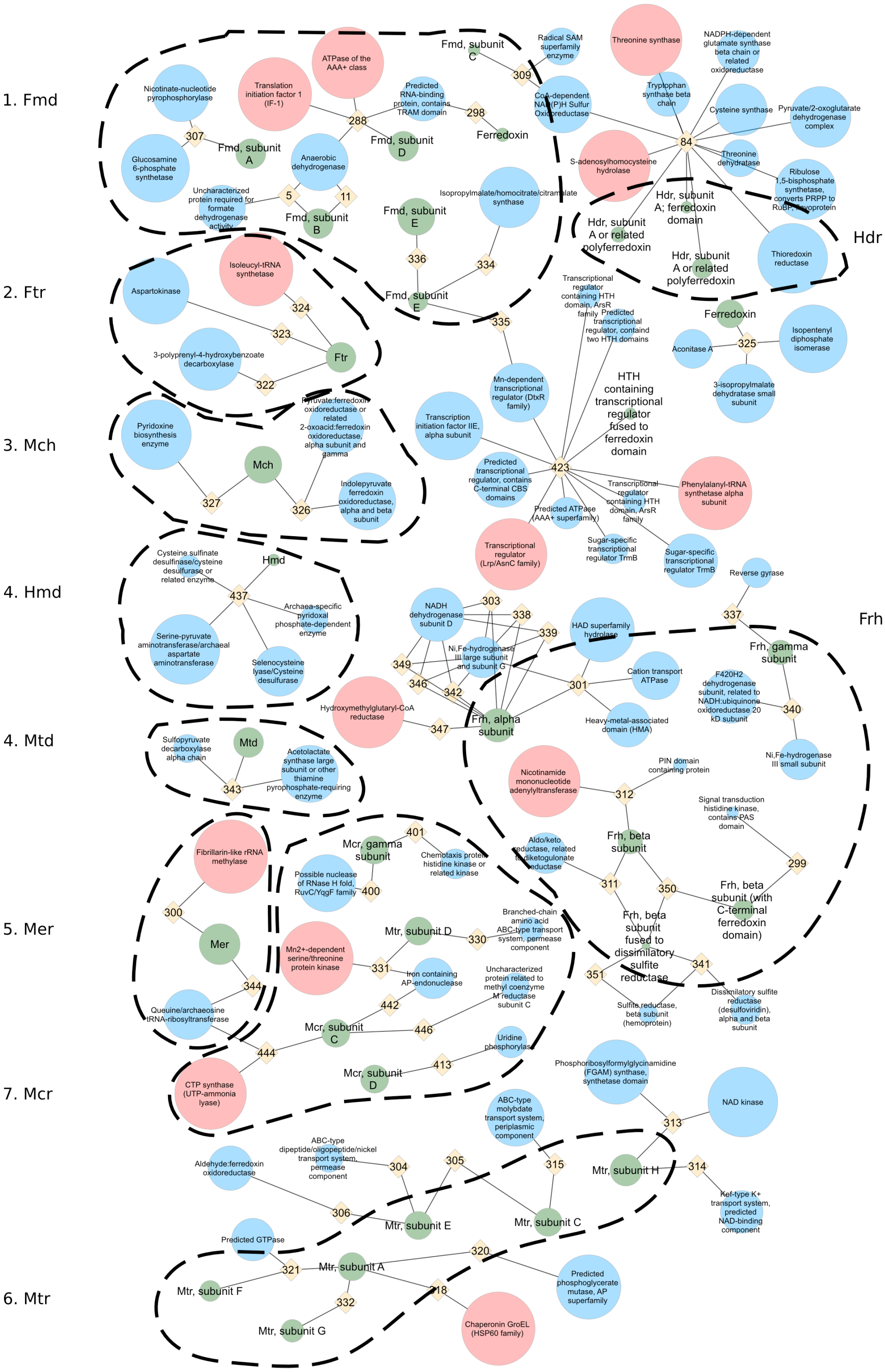

Figure S1. **Graph of connections between methanogenesis-related arCOGs and profiles of EFLs**

Circular nodes represent arCOGs and the size of the node represents the number of archaeal lineages for each arCOG. Core arCOGs are colored red, shell is blue, and arCOGs involved in methanogenesis are green. Sequence profiles of the elementary functional loops are represented as orange diamonds in the graph. The edges between the diamonds and the circles represent profile-arCOG matches. The dashed lines show the groups of arCOGs corresponding to enzymes in each step of the methanogenic pathway.

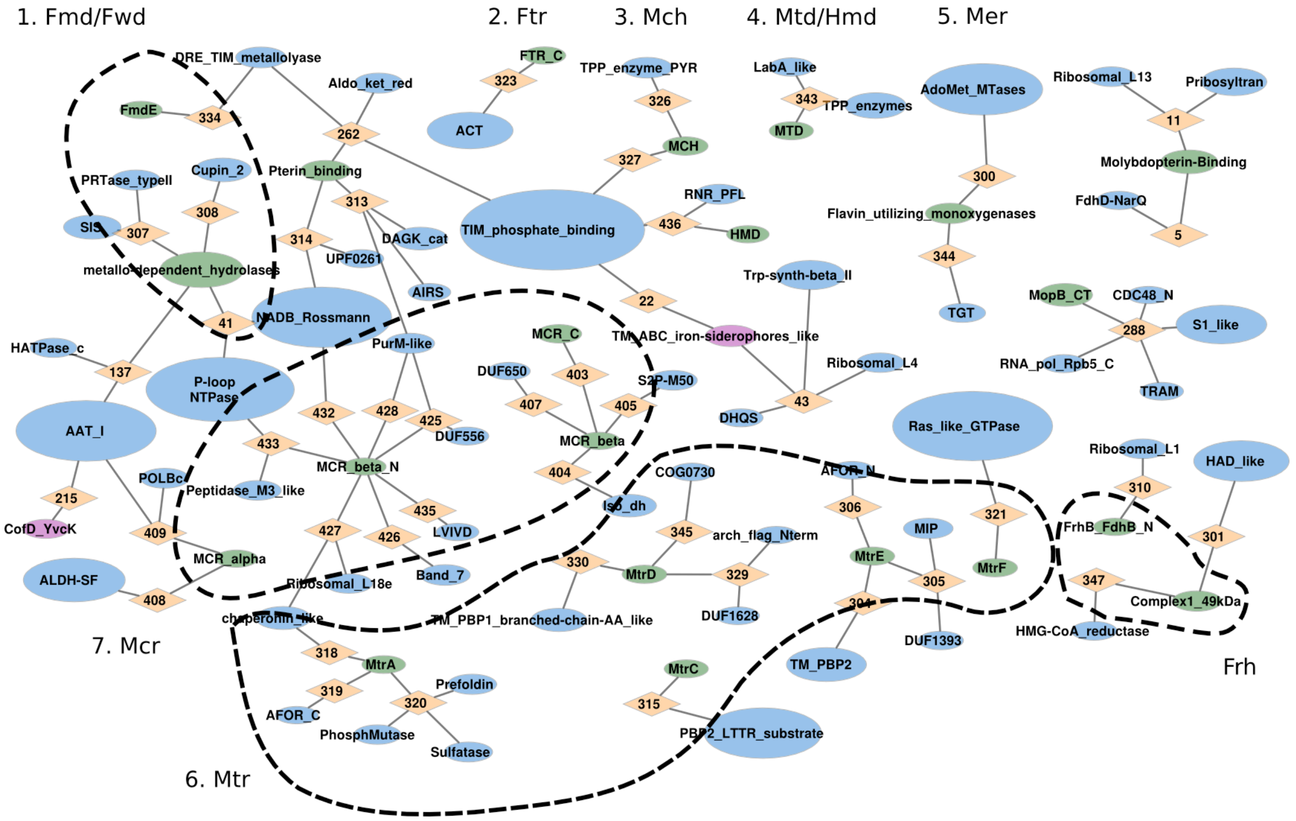

Figure S2. Graph of connections between methanogenesis-related CDD domains and profiles of EFLs

Connections between sequence profiles (orange diamonds) and CDD superfamilies (oval nodes) represent matches between sequence profiles and the sequences of CDD domains. Superfamilies associated with methanogenesis are colored green. The ones involved in cofactor biosynthesis are colored magenta. The size of the node represents the number of families in the corresponding CDD superfamily.
