# Supplementary material for: Exploring the evolution of protein function in Archaea
Source: BMC Evol Biol. 2012 May 30;12:75. doi: 10.1186/1471-2148-12-75 (PMC3458885; doi:10.1186/1471-2148-12-75)
Supplement: Additional file 4 — Contains the list of archaeal proteomes used to obtain sequence profiles. [file 1471-2148-12-75-S4.pdf]

Exploring the evolution of protein function in Archaea

Alexander Goncearenco and Igor N. Berezovsky

Additional File 4

Table ST4. Archaeal proteomes used to converge the prototypes of EFLs
The following 68 archaeal proteomes were used to derive sequence prototypes. Four proteomes (bold font), each representing a separate archaeal phylum, were used as source of origins.

Methanococcus maripaludis C7
Candidatus Methanoregula boonei 6A8
Caldivirga maquilingensis IC-167
Methanococcus maripaludis C5
Methanothermobacter thermautotrophicus str Delta H
Staphylothermus marinus F1
Haloarcula marismortui ATCC 43049
Methanococcus aeolicus Nankai-3
Nitrosopumilus maritimus SCM1
Thermoproteus neutrophilus V24Sta
Thermococcus kodakarensis KOD1
Pyrobaculum islandicum DSM 4184
Methanococcus vannielii SB
Sulfolobus islandicus L S 2 15
Sulfolobus islandicus Y N 15 51
Halorhabdus utahensis DSM 12940
Ignicoccus hospitalis KIN4
Ignicoccus hospitalis KIN4/I
Methanoculleus marisnigri JR1
Sulfolobus solfataricus P2
Methanospirillum hungatei JF-1
Sulfolobus tokodaii str 7
Methanosaeta thermophila PT
Halobacterium sp NRC-1
Methanosphaerula palustris E1-9c
Archaeoglobus fulgidus DSM 4304
Picrophilus torridus DSM 9790
Aeropyrum pernix K1
Thermoplasma acidophilum DSM 1728
Desulfurococcus kamchatkensis 1221n
Thermococcus sibiricus MM 739
Methanosphaera stadtmanae DSM 3091
Sulfolobus islandicus M 16 4
Pyrobaculum arsenaticum DSM 13514
Methanobrevibacter smithii ATCC 35061
Methanosarcina acetivorans C2A
Pyrococcus horikoshii OT3
Sulfolobus islandicus M 16 27
Halomicrobium mukohataei DSM 12286
Natronomonas pharaonis DSM 2160
Candidatus Korarchaeum cryptofilum OPF8
uncultured methanogenic archaeon RC-I
Pyrococcus furiosus DSM 3638
Thermoplasma volcanium GSS1
Halorubrum lacusprofundi ATCC 49239
Halobacterium salinarum R1
Methanosarcina mazei Go1
Pyrobaculum aerophilum str IM2
Methanopyrus kandleri AV19
Methanosarcina barkeri str Fusaro
Methanocorpusculum labreanum Z
Sulfolobus islandicus Y G 57 14
Pyrococcus abyssi GE5
Methanococcus maripaludis C6
Methanococcoides burtonii DSM 6242
Methanococcus maripaludis S2
Haloquadratum walsbyi DSM 16790
Thermococcus gammatolerans EJ3
Sulfolobus acidocaldarius DSM 639
Metallosphaera sedula DSM 5348
Thermofilum pendens Hrk 5
Methanocaldococcus fervens AG86
Hyperthermus butylicus DSM 5456
Pyrobaculum calidifontis JCM 11548
Nanoarchaeum equitans Kin4-M
Sulfolobus islandicus M 14 25
Thermococcus onnurineus NA1
Methanocaldococcus jannaschii DSM 2661
